# Polyaspartic Acid‐Calcium‐Lanthanum Complexes Induce Antibacterial Remineralization of Dentin and In‐Depth Occlusion of Dentinal Tubules

**DOI:** 10.1002/advs.202501340

**Published:** 2025-04-27

**Authors:** Ling Zhu, Wei Liu, Yizhou Zhang, Zhifang Wu, Yiru Wang, Yuedan Xu, Haiyan Zheng, Hongli Zhang, Mengfei Yu, Xiaoting Jin, Zhe Wang, Zihuai Zhou, Baiping Fu

**Affiliations:** ^1^ Department of Stomatology Children's Hospital Zhejiang University School of Medicine National Clinical Research Center for Child Health Hangzhou Zhejiang 310000 China; ^2^ Stomatology Hospital School of Stomatology Zhejiang University School of Medicine Zhejiang Provincial Clinical Research Center for Oral Diseases Key Laboratory of Oral Biomedical Research of Zhejiang Province Engineering Research Center of Oral Biomaterials and Devices of Zhejiang Province Cancer Center of Zhejiang University Hangzhou Zhejiang 310000 China

**Keywords:** antibacterial, dental caries, dentinal tubule occlusion, intrafibrillar mineralization, polyaspartic acid‐calcium‐lanthanum complexes

## Abstract

Dental caries is a global oral dilemma leading to demineralization of tooth hard tissues and exposure of dentinal tubules (DTs). Multifunctional restorative strategies, including remineralization, antibacterial properties, and DT occlusion, are urgently needed for dental caries management but remain a significant challenge. Herein, the polyaspartic acid‐calcium‐lanthanum suspension (5 g L^−1^‐3.06 м‐0.34 м) followed by phosphate solution (2.04 м) is adopted to conduct polyelectrolyte‐cation complexes pre‐precursor process. This strategy, for the first time, successfully induces La‐doped intrafibrillar mineralization of collagen fibrils with lanthanum‐doped hydroxyapatite (La‐HAp). After sequential application of two mineralization media, the demineralized dentin is fully remineralized and the DTs are deeply occluded to a depth of 100 µm with La‐HAp after 7 d of incubation in artificial saliva or oral cavity of rabbit. The remineralized dentin demonstrates antibacterial properties against cariogenic bacteria (*Streptococcus mutans*) both in vitro and in vivo, and its mechanical properties are almost restored to those of intact dentin. The mineralization media cause no apparent irritation to oral mucosa or dental pulp. Hence, this multifunctional strategy confers the remineralized dentin surface with antibacterial properties and blocks the invasion of cariogenic bacteria through DTs, providing insight into the clinical management of dentin caries accompanied by dentin hypersensitivity.

## Graphical abstract

Polyaspartic acid‐calcium‐lanthanum (PAsp‐Ca‐La) complexes could induce antibacterial remineralization of demineralized dentin and in‐depth occlusion of dentinal tubules (DTs) with lanthanum‐doped hydroxyapatite (La‐HAp) via polyelectrolyte‐cation complexes pre‐precursor (PCCP) process, allowing the release of La^3+^ in response to acids generated by *Streptococcus mutans* and inhibiting bacterial invasion through DTs. This multifunctional strategy might be a promising approach for management of dentin caries.

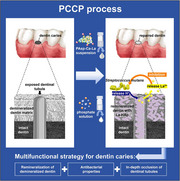

## 1 Introduction

Dental caries, a global oral disease, affects over 3.5 billion individuals worldwide, posing an enormous socioeconomic burden.^[^ ^1^, ^2^ ^]^ In most instances, owing to the metabolite acids generated by cariogenic bacteria, dental caries tends to cause a rapid dental tissue defect and exposure of dentinal tubules (DTs).^[^ ^3^, ^4^ ^]^ The cariogenic bacteria are likely to invade deep dentinal tissues through the exposed DTs, thereby further accelerating the progression of dentin caries.^[^ ^5^, ^6^ ^]^ Therefore, the remineralization of demineralized dentin matrix (DDM) with antibacterial properties, as well as the in‐depth occlusion of DTs, are equally crucial in the initial stage of dentin caries progression, which still remains a great challenge.^[^ ^7^, ^8^, ^9^ ^]^

Numerous strategies have been proposed to achieve deep occlusion of DTs, including the application of hydrogels to block the fluid flow and directional cation transport within DTs, yet often neglecting the dentin remineralization and antibacterial properties.^[^ ^10^, ^11^ ^]^ Li et al. reported that an effective anti‐fouling dentin surface was constructed using amyloid‐inspired surface modification to resist the adhesion of *Streptococcus mutans* (*S. mutans*) and induce hydroxyapatite (HAp) deposition within the DTs.^[^ ^6^ ^]^ Hitherto, the polymer‐induced liquid‐precursor (PILP) process is the most acknowledged mineralization strategy for the remineralization of DDM in vitro,^[^ ^12^, ^13^, ^14^, ^15^, ^16^, ^17^, ^18^, ^19^, ^20^, ^21^ ^]^ with only a certain ability to occlude DTs and no antibacterial properties.^[^ ^22^ ^]^ As the key of the PILP process, amorphous calcium phosphate (ACP) precursors could infiltrate into the collagen fibrils via capillary action,^[^ ^12^, ^13^ ^]^ electrostatic attraction,^[^ ^23^, ^24^ ^]^ or Gibbs‐Donnan equilibrium^[^ ^25^ ^]^ for inducing intrafibrillar mineralization. However, these precursors tend to crystallize rapidly even when they are stabilized by polyelectrolytes in a low‐concentration solution, thus losing their mineralization activity.^[^ ^16^, ^17^ ^]^ Therefore, the mineralization medium needs to be continuously refreshed. This is a great shortcoming of clinical applications. In a word, an effective strategy for management of dentin caries demands multifunctional manipulation of dentin remineralization, antibacterial effects, and occlusion of DTs, which still requires further exploration.^[^ ^7^, ^8^, ^9^ ^]^

Recently, the polyelectrolyte‐cation complexes pre‐precursor (PCCP) process has been demonstrated to be a promising strategy for the management of dentin caries and dentin hypersensitivity.^[^ ^26^, ^27^, ^28^ ^]^ Briefly, the electropositive polyaspartic acid‐calcium (PAsp‐Ca) complexes rapidly penetrate into the electronegative collagen fibrils, DDM, and DTs via electrostatic attraction, capillary action and osmotic pressure, and subsequently the phosphate groups are attracted to achieve the complete remineralization of 100‐120‐um dentin artificial caries lesions and in‐depth occlusion of DTs over 150–200 µm.^[^ ^26^, ^27^, ^28^ ^]^ To endow the dentin surface with antibacterial properties, a strontium‐ or magnesium‐doped (Sr/Mg‐doped) remineralized dentin layer was constructed via PCCP process using polyaspartic acid‐strontium (PAsp‐Sr) or polyaspartic acid‐calcium‐magnesium (PAsp‐Ca‐Mg) complexes.^[^ ^29^, ^30^ ^]^ However, the co‐doping with fluorine ion (F^−^) or pre‐treatment of collagen fibrils with citrate is essential to eliminate the Sr^2+^/Mg^2+^‐induced inhibition in the nucleation of HAp crystals.^[^ ^31^, ^32^, ^33^, ^34^, ^35^ ^]^ Moreover, both the incorporation of Sr^2+^ or Mg^2+^ into HAp could indeed reduce the structural stability of HAp.^[^ ^36^, ^37^, ^38^ ^]^

**Figure 1:**
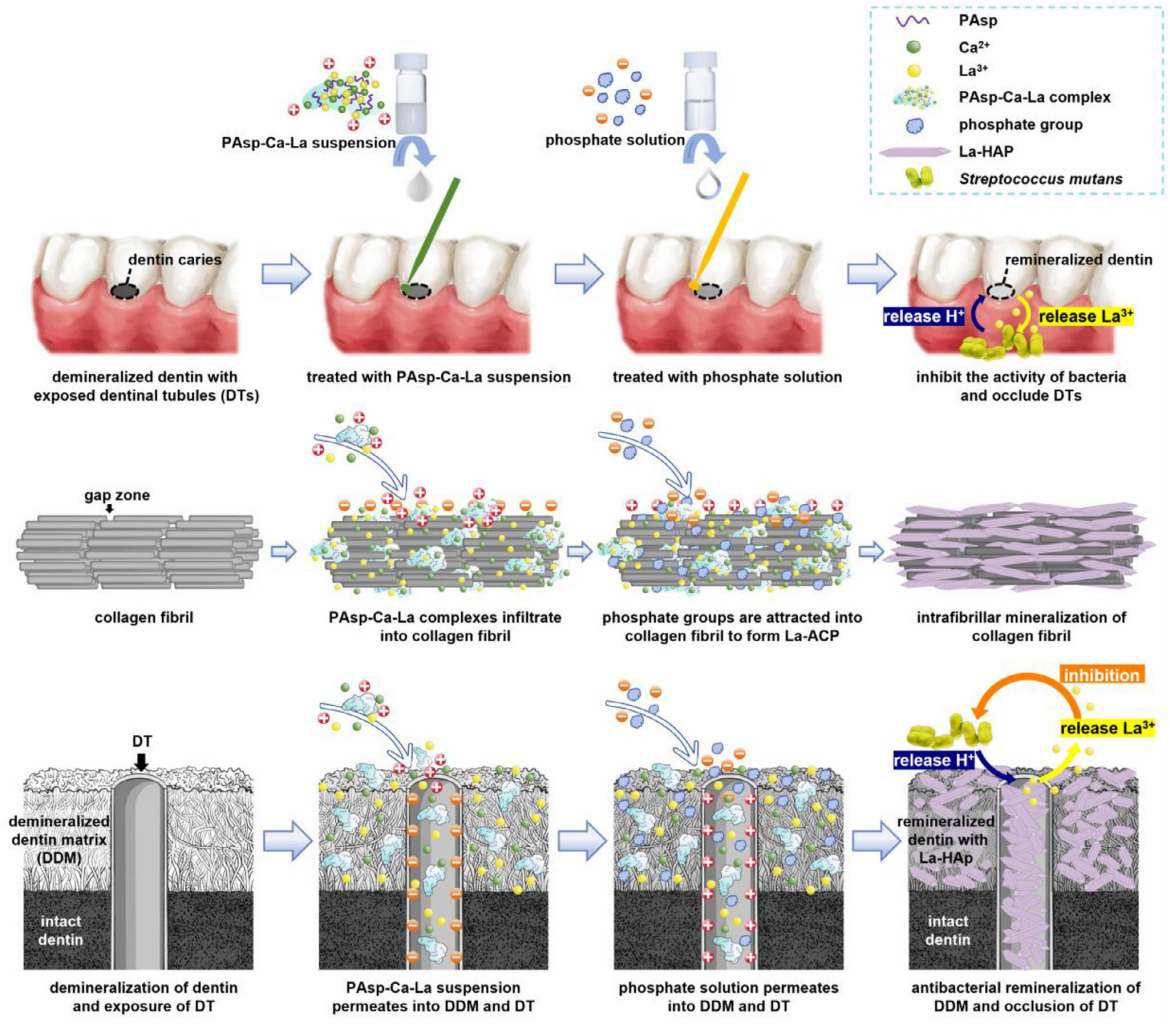
Diagram of the remineralization of DDM with antibacterial properties and in‐depth occlusion of DTs via PCCP process. After the DDM with exposed DTs is sequentially treated with PAsp‐Ca‐La suspension and phosphate solution, the electropositive PAsp‐Ca‐La complexes along with free Ca^2+^ and La^3+^ ions infiltrate into the electronegative collagen fibrils, DDM, and DTs via electrostatic attraction, capillary action and osmotic pressure, and subsequently the phosphate groups are attracted to form lanthanum‐doped amorphous calcium phosphate (La‐ACP). In artificial saliva or oral cavity, the La‐ACP precursors progressively crystallize into La‐HAp. This La‐doped mineralization strategy could achieve antibacterial remineralization of DDM and in‐depth occlusion of DTs with La‐HAp, allowing the release of La^3+^ ions in response to the acids generated by cariogenic bacteria (*Streptococcus mutans*) and inhibiting bacterial invasion through DTs.

As one of the most prominent rare earth elements, lanthanum ion (La^3+^) has been extensively used to substitute calcium ion (Ca^2+^) of HAp, owing to the similar atomic radius and chemical characteristics.^[^ ^39^, ^40^, ^41^ ^]^ Numerous studies have investigated lanthanum‐doped hydroxyapatite (La‐HAp) as the bone graft materials or implant coatings, due to its superior biocompatibility, osteoinductive and antibacterial properties.^[^ ^42^, ^43^, ^44^, ^45^, ^46^ ^]^ Moreover, in contrast to the doping of Sr^2+^ and Mg^2+^, the incorporation of La^3+^ into the HAp crystal lattice could improve its crystallinity and acid resistance, which might be attributed to the excess energy due to higher valance of La^3+^ than Ca^2+^.^[^ ^42^, ^43^, ^44^, ^45^ ^]^ Herein, we proposed a PCCP process using polyaspartic acid‐calcium‐lanthanum (PAsp‐Ca‐La) complexes as pre‐precursors in absence of fluorine or modification of collagen fibrils. This strategy successfully induced the La‐doped intrafibrillar mineralization of collagen fibrils with La‐HAp for the first time (**Figure** **1**). Furthermore, a La‐doped remineralized dentin layer was constructed and the DTs were deeply occluded over 100 µm with La‐HAp (Figure 1), conferring the remineralized dentin surface with antibacterial properties and blocking the invasion of cariogenic bacteria through DTs. This multifunctional strategy might be a promising approach for the clinical management of dentin caries, particularly when accompanied by dentin hypersensitivity.

## 2 Results and Discussion

### 2.1 Characterization of PAsp‐Ca‐La Complexes

**Figure 2:**
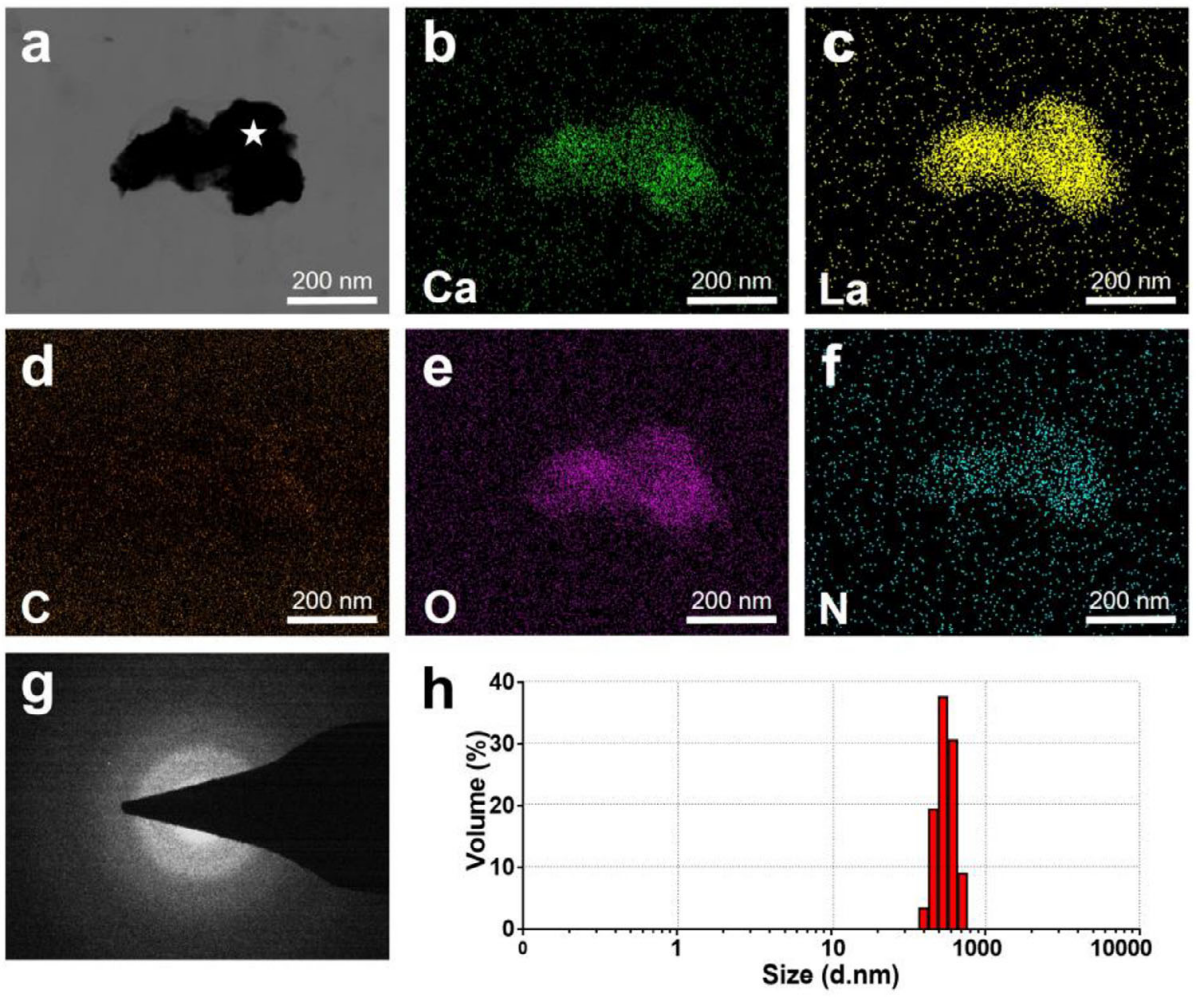
Characterization of the PAsp‐Ca‐La complexes. a–g) STEM image (a) with elemental mapping (b–f) and SAED pattern (g) shows an irregular morphology of PAsp‐Ca‐La complexes as an amorphous entity, containing calcium (b), lanthanum (c), carbon (d), oxygen (e) and nitrogen (f) elements. The SAED pattern (g) was obtained from the point (white pentagram) in (a). h) The DLS result indicated that the mean diameter of PAsp‐Ca‐La complexes was ≈497.9 ± 90.65 nm.

In this study, the PCCP mineralization media included PAsp‐Ca‐La suspension (5 g L^−1^‐3.06 м‐0.34 м) and phosphate solution (2.04 м). Wherein, PAsp‐Ca‐La complexes were formed in the PAsp‐Ca‐La suspension. Representative scanning transmission electron microscopy (STEM) image (**Figure** **2**a) shows an irregular shape of PAsp‐Ca‐La complexes with a high electron density.^[^ ^47^, ^48^ ^]^ The elemental mapping results (Figure 2b–f) showed that the PAsp‐Ca‐La complexes contain numerous calcium and lanthanum elements, as well as nitrogen elements from PAsp molecules. The selected area electron diffraction (SAED) pattern (Figure 2g) indicates an amorphous phase of the PAsp‐Ca‐La complexes. Although the stoichiometric La/(Ca+ La) ratio was set to 0.1 in suspension, the ratio of the formed PAsp‐Ca‐La complexes was much higher as 0.48 ± 0.06. The discrepancy is because the trivalent La^3+^ has a stronger affinity with carboxyl groups of PAsp molecules than the bivalent Ca^2+^.^[^ ^47^, ^48^ ^]^ The dynamic light scattering (DLS) result revealed an average diameter of ≈497.9 ± 90.65 nm of PAsp‐Ca‐La complexes (Figure 2h). This is consistent with the STEM image (Figure 2a). Due to a great amount of free Ca^2+^ and La^3+^ ions, PAsp‐Ca‐La complexes exhibited an electropositive zeta potential of +6.05 ± 3.14 mV.^[^ ^26^ ^]^ The electropositivity and amorphous structure of PAsp‐Ca‐La complexes indicate a potential to induce remineralization of DDM and occlusion of DTs via the PCCP process.^[^ ^26^, ^27^ ^]^

### 2.2 La‐Doped Intrafibrillar Mineralization of Collagen Fibrils

**Figure 3:**
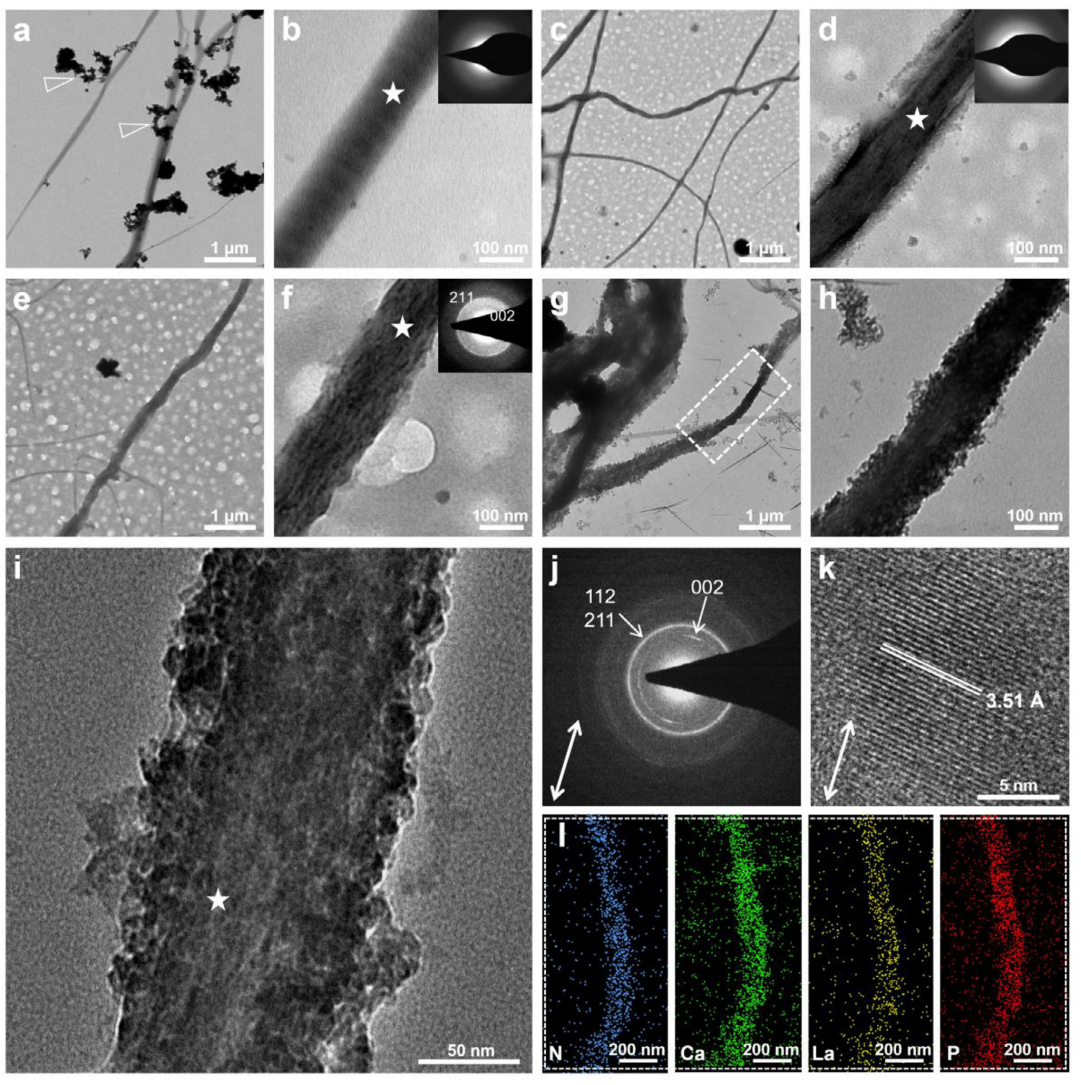
TEM and HRTEM images with SAED patterns and elemental mapping of the La‐doped intrafibrillar mineralization of collagen fibrils via PCCP process. a,b) TEM images with SAED pattern show that the collagen fibrils turned darker after 1‐h treatment with PAsp‐Ca‐La suspension, and attached with several PAsp‐Ca‐La complexes (white arrowheads in (a)). c,d) TEM images with SAED pattern show that electron density of collagen fibrils was increased and amorphous minerals were generated after treatment with phosphate solution for 1 h. e,f) TEM images with SAED pattern show the partial mineralization of collagen fibrils with faint crystals and weak arcs of the (002) and (211) planes after 1 d of incubation in artificial saliva. g–k) TEM (g,h) and HRTEM images with SAED pattern (i–k) show that the collagen fibrils were completely mineralized with distinct arcs of the (002), (211), and (112) and a d‐spacing of 3.51 Å after 4 d of incubation in artificial saliva. The c‐axis of the crystals on the collagen fibrils (j,k) is almost in parallel with the long axis of the collagen fibril in (i). The white bidirectional arrows in (j,k) represent the long axis of collagen fibril in (i). The SAED patterns in (b,d,f,j) were obtained from the points marked with white pentagrams in (b,d,f,i), respectively. l) The elemental mapping of mineralized fibril in the white line box area in (g) indicates the even distribution of nitrogen, calcium, lanthanum, and phosphorus elements on the collagen fibril.

This study further confirms the hypothesis of the PCCP process as partial substitution of Ca^2+^ by La^3+^. The sequential treatment with PAsp‐Ca‐La suspension (5 g L^−1^‐3.06 м‐0.34 м) and phosphate solution (2.04 м) each for 1 h, followed by 4 d of incubation in artificial saliva, successfully induced the complete intrafibrillar mineralization of collagen fibrils with La‐HAp (**Figure** **3**; Figure S1, Supporting Information).

After treatment with PAsp‐Ca‐La suspension for 1 h, the TEM and high‐resolution transmission electron microscopy (HRTEM) images with SAED patterns and elemental mapping show an evident increase of electron density of collagen fibrils (Figure 3a,b; Figure S1a,b, Supporting Information), indicating that PAsp‐Ca‐La complexes along with numerous Ca^2+^ and La^3+^ ions entered the collagen fibrils by virtue of electrostatic attraction and osmotic pressure.^[^ ^26^ ^]^ In particular, although the diameter of PAsp‐Ca‐La complexes (497.9 ± 90.65 nm, Figure 2) greatly exceeds the width of gap zone (40 nm),^[^ ^49^ ^]^ these complexes could still infiltrate into the intrafibrillar spaces owing to their liquid‐like properties (Figure S1a,b, Supporting Information). This is consistent with our previous publication about the PAsp‐Ca complexes.^[^ ^26^ ^]^ In addition, the attenuated total reflectance Fourier transform infrared spectroscopy (ATR‐FTIR) spectra reveal that the peak of amide I band at 1633 cm^−1^ downshifts to 1629 cm^−1^, illustrating the chemical chelation between carbonyl groups of collagen fibrils and the Ca^2+^ or La^3+^ (Figure S2a, Supporting Information).^[^ ^50^, ^51^ ^]^

After subsequent treatment with phosphate solution for 1 h, some Ca^2+^ and La^3+^ ions ran out of the collagen fibrils at first, however, the majority of PAsp‐Ca‐La complexes were firmly anchored within collagen fibrils by chemical chelation and phosphate groups were further attracted, resulting in the uneven distribution of calcium, lanthanum and phosphorus elements within the collagen fibrils (Figure S1c, Supporting Information). As more phosphate groups entered the collagen fibrils to react with Ca^2+^ and La^3+^ ions to form lanthanum‐doped amorphous calcium phosphate (La‐ACP), a uniform distribution of calcium, and lanthanum and phosphorus elements within the collagen fibrils was ultimately obtained (Figure 3c,d; Figure S1d, Supporting Information).

After the collagen fibrils were incubated in artificial saliva, the minerals gradually transformed from an amorphous phase (Figure 3d; Figure S1d, Supporting Information) to crystals after 4 d (Figure 3e–l; Figure S1e, Supporting Information). The c‐axis of the crystals is almost parallel to the long axis of the collagen fibril (Figure 3i–k; Figure S1e, Supporting Information). This indicates intrafibrillar mineralization.^[^ ^14^, ^52^, ^53^ ^]^ The HRTEM images with SAED pattern of mineralized collagen fibrils (Figure 3j,k; Figure S1e, Supporting Information) and the ATR‐FTIR spectra of mineralized gels (Figure S2b, Supporting Information) reveal that the generated crystals are similar to HAp with slight discrepancies in the interplanar spacings and peak values.^[^ ^42^, ^45^ ^]^ The incorporation of La^3+^ (1.016 Å) with a larger ionic radius than Ca^2+^ (0.99 Å) into HAp lattices is attributed to the increased d‐spacing of (002) plane from 3.44 Å of HAp to 3.51Å (Figure 3k).^[^ ^42^, ^43^, ^44^ ^]^

Notably, the variation of La/(Ca + La) and (Ca + La)/P ratios of the collagen fibrils reveals the intrafibrillar crystallization of La‐HAp crystals (Figure S3, Supporting Information). Initially, the La/(Ca + La) ratio of the collagen fibrils was 0.48 ± 0.04 after the treatment with PAsp‐Ca‐La suspension (Figure S3a,d, Supporting Information). After subsequent treatment with phosphate solution, the La/(Ca + La) and (Ca + La)/P ratios of collagen fibrils were 0.49 ± 0.08 and 0.40 ± 0.06 respectively (Figure S3b,d, Supporting Information), indicating the influx of phosphate groups and the formation of La‐ACP. After the complete mineralization of collagen fibrils, the La/(Ca + La) ratio decreased to 0.23 ± 0.09 (Figure S3c,d, Supporting Information), indicating the outflow of some La^3+^ ions during crystallization of La‐HAp.^[^ ^54^ ^]^ Moreover, the (Ca + La)/P ratio dramatically increased to 1.53 ± 0.15 (Figure S3c,d, Supporting Information), slightly lower than 1.67 of HAp due to the higher electropositivity of La^3+^ than Ca^2+^.^[^ ^55^ ^]^ These stoichiometric fluctuations are consistent with the crystallization process of La‐HAp outside the collagen fibrils (Figure S4, Supporting Information).

Conversely, the PILP process, as a recognized strategy for the mineralization of collagen fibrils with HAp, has seldom been proposed for ions‐doped intrafibrillar mineralization.^[^ ^31^, ^32^, ^33^ ^]^ This study synthesized the PAsp‐stabilized La‐ACP to induce La‐doped intrafibrillar mineralization (Figure S5a–c, Supporting Information). The ratios of La/(Ca + La) and (Ca + La)/P for the synthesized La‐ACP were 0.03 and 1.02 respectively, indicating the limited incorporating capacity of La^3+^ into ACP despite the La/(Ca + La) ratio of the mineralization medium being set to 0.1 (Figure S5c, Supporting Information). After 4 d of treatment with PAsp‐stabilized La‐ACP solution, only segmental intrafibrillar mineralization of collagen fibrils was detected (Figure S5d–f, Supporting Information). The ratios of La/(Ca + La) and (Ca + La)/P for the mineralized region of collagen fibrils were 0.04 ± 0.02 and 1.10 ± 0.35, respectively (Figure S5d,f, Supporting Information), which are significantly lower than ratios of La/(Ca + La) (0.23 ± 0.09) and (Ca + La)/P (1.53 ± 0.15) via the PCCP process (*p* < 0.05, Figure S3c,d, Supporting Information). HRTEM images with SAED patterns show that La‐ACP tends to crystallize more quickly than ACP in solution (Figure S6, Supporting Information). In a word, both the La‐doped PILP and PCCP process could achieve the intrafibrillar mineralization of collagen fibrils with La‐HAp (Figure 3; Figures S1, S5, Supporting Information). Since La^3+^ could promote crystallization of La‐ACP to La‐HAp outside collagen fibrils, this accounts for the poor La‐doped intrafibrillar mineralization via PILP process (Figure S5d–f, Supporting Information).^[^ ^56^ ^]^ On the contrary, the La‐doped mineralization via the PCCP process could benefit from the instability of La‐ACP, because the La‐ACP was formed within collagen fibrils and rapidly transformed to La‐HAp once phosphate groups successively entered the collagen fibrils following the penetration of PAsp‐Ca‐La complexes along with free Ca^2+^ and La^3+^ ions (Figure 3; Figure S1, Supporting Information).^[^ ^26^ ^]^ Moreover, the characteristics of La^3+^ in promoting the crystallization of La‐HAp exhibit its superior to substitutions of Sr^2+^ and Mg^2+^ in intrafibrillar mineralization via PCCP process,^[^ ^29^, ^30^ ^]^ especially in the absence of pre‐treatment of collagen fibrils with citrate and assistance of fluorine to counteract the inhibitory effect of nucleation of Sr^2+^ and Mg^2+^.^[^ ^31^, ^32^, ^33^, ^34^, ^35^ ^]^

Particularly, PAsp‐Ca‐La complexes play a crucial role in the La‐doped intrafibrillar mineralization via PCCP process, which was confirmed by the negative consequences when the Ca‐La suspension (3.06 м–0.34 м) was employed in replace of the PAsp‐Ca‐La suspension (Figure S7, Supporting Information). Once the phosphate solution (2.04 м) was subsequently applied, La‐ACP was generated outside the collagen fibrils (Figure S7a–e, Supporting Information). In the absence of PAsp, the La‐ACP transformed into La‐HAp in interfibrillar spaces, leaving poor intrafibrillar mineralization of collagen fibrils (Figure S7f,g, Supporting Information).

### 2.3 In Vitro and In Vivo Remineralization of DDM and Occlusion of DTs

**Figure 4:**
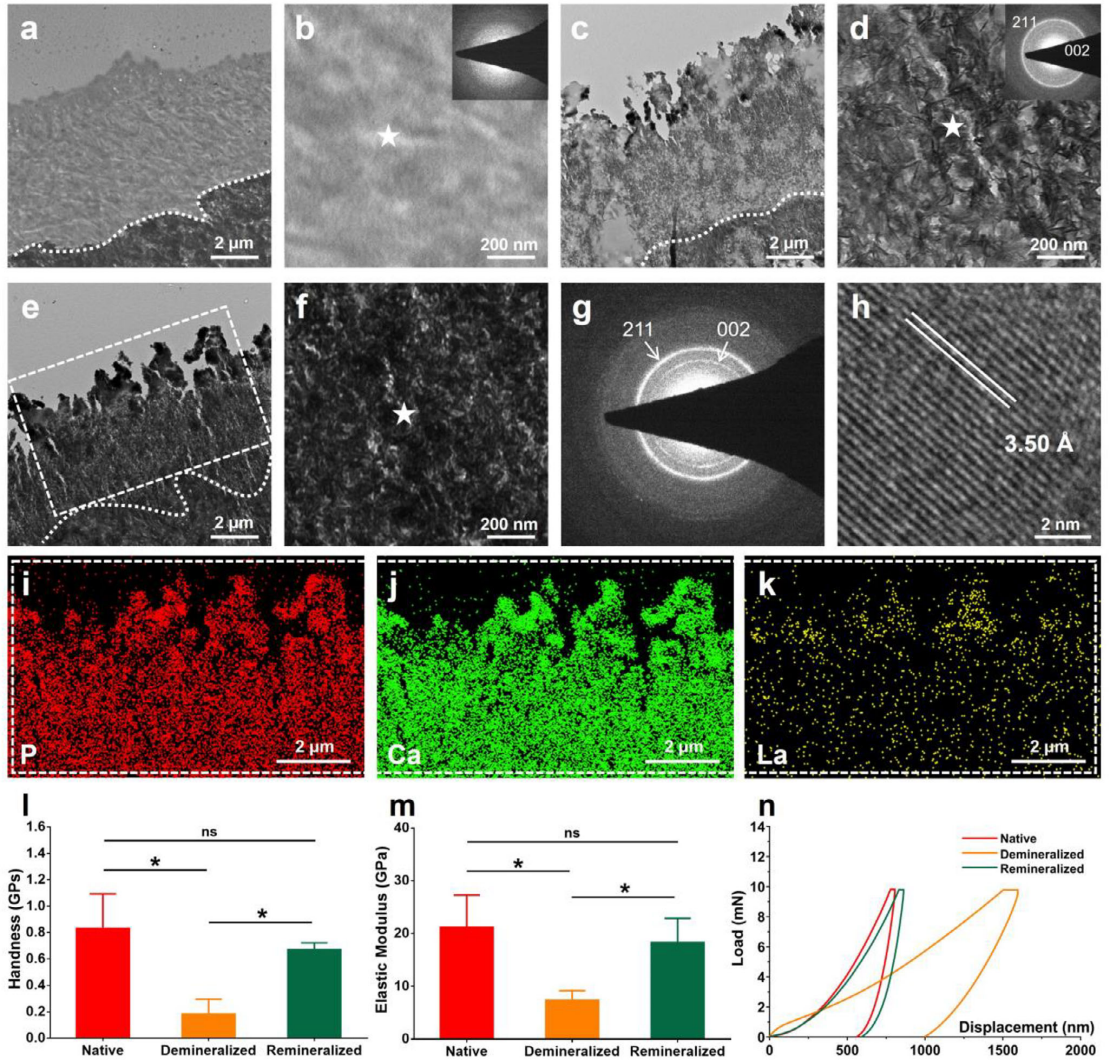
TEM, HRTEM images, and nanoindentation of remineralized dentin via PCCP process. a,b) TEM images with SAED pattern of the demineralized dentin show a demineralized layer with the thickness of 4–6 µm. c,d) TEM images with SAED pattern of the remineralized dentin indicate an incomplete remineralization consisting of crystallites with weak typical (002) and (211) planes after the sequential treatment with PAsp‐Ca‐La suspension followed by phosphate solution, and incubation in the artificial saliva for 1 d. e,f) TEM images of the remineralized dentin display complete remineralization after 7 d of incubation in artificial saliva. g,h) The SAED pattern (g) and HRTEM image (h) exhibit the crystals with typical (002) and (211) planes and a d‐spacing of 3.50 Å in the remineralized dentin. The SAED patterns in (b,d,g) were obtained from the points marked with white pentagrams in (b,d,f), respectively. i–k) The elemental mapping of the remineralized dentin (white dotted box in (e)) indicates the distribution of the phosphorus, calcium, and lanthanum elements in the remineralized layer. The La/(Ca + La) ratio of the remineralized layer was 0.11 ± 0.05. l–n). The mechanical properties including hardness (l) and elastic modulus (m) of the native dentin, demineralized dentin, and remineralized dentin, were calculated from the load‐displacement curves (n). The data of hardness (l) and elastic modulus (m) are presented as mean ± SD, *n* = 3, and one‐way ANOVA testing followed by a Tukey post‐hoc test was carried out across groups. “*” indicates significant difference (*p* < 0.05) and “ns” indicates no significant difference (*p* > 0.05).

In the clinical scenario, cariogenic bacteria produce acid to cause demineralization of dentin and exposure of DTs.^[^ ^3^, ^4^ ^]^ On one hand, the DDM suffers from the enzymolysis of collagenase and leads to tooth‐hard tissue defects.^[^ ^3^ ^]^ On the other hand, the open DTs facilitate the invasion of bacteria in caries progression and result in dentin hypersensitivity.^[^ ^4^, ^5^, ^6^ ^]^ Hence, the occlusion of DTs and remineralization of the involved dentin matrix with antibacterial properties are equally of vital importance in the management of dentin caries.^[^ ^7^, ^8^, ^9^ ^]^ In this study, the human dentin disk and New Zealand rabbit incisor dentin were etched with 37% phosphoric acid to generate a layer of DDM ≈4–6 µm in thickness (**Figure** **4**a,b) and extensively exposed DTs (Figure S8, Supporting Information), respectively, for in vitro and in vivo experiments.

**Figure 5:**
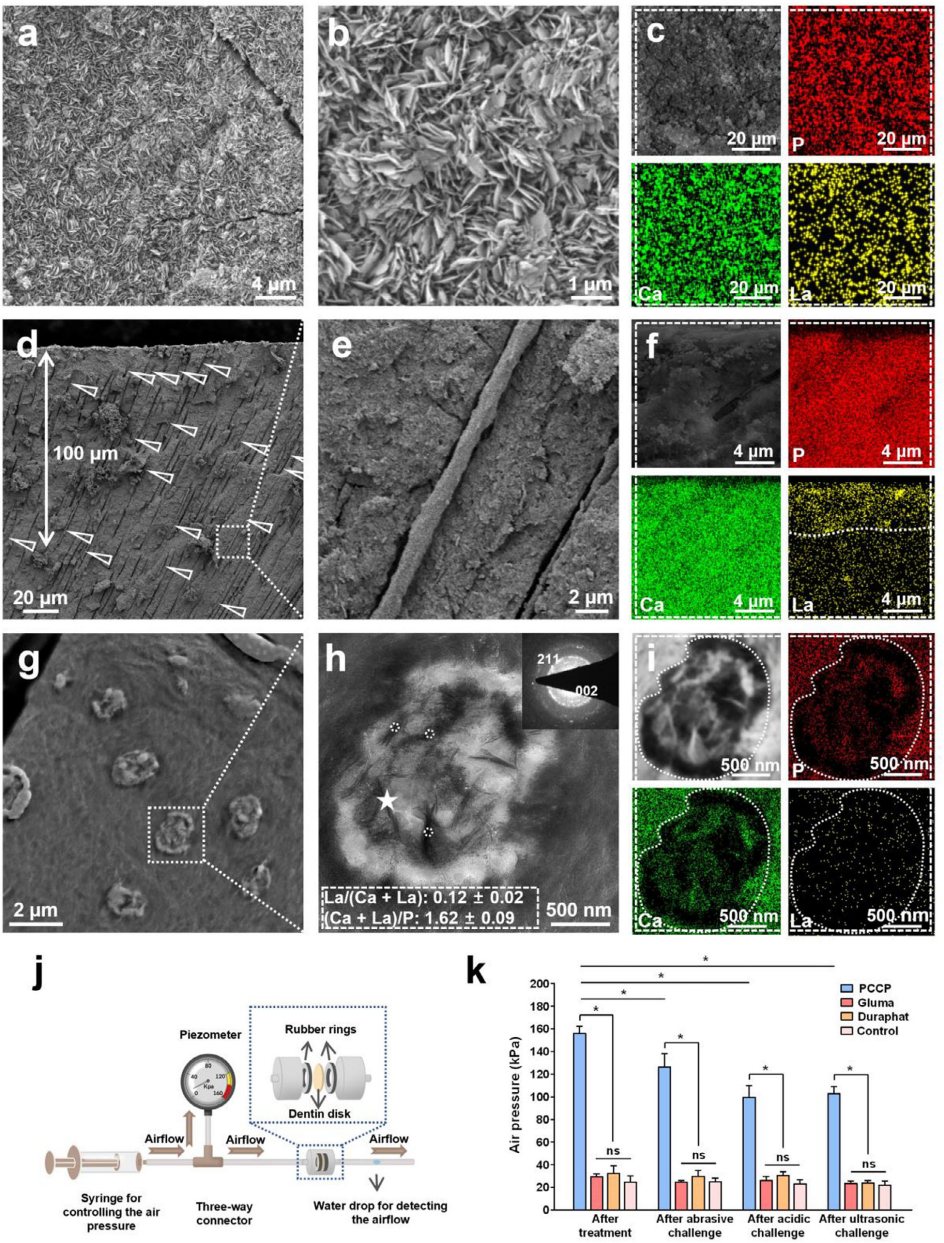
The occlusion of DTs via PCCP process. a,b) SEM findings of remineralized dentin surface indicated that a compact layer of crystals was deposited on the dentin surface and the DTs were fully covered. c) SEM finding with elemental mapping revealed that these crystals were composed of phosphorus, calcium, and lanthanum elements. d,e) SEM findings of the longitudinal sections of the remineralized dentin indicated that the DTs were deeply occluded by dense crystals up to 100 µm. The white arrowheads in (d) represent the occluding minerals with DTs. f) SEM finding with elemental mapping showed the distribution of calcium, lanthanum, and phosphorus elements in the superficial remineralized dentin. The phosphorus and calcium elements were evenly distributed from the remineralized dentin to the underlying intact dentin, whereas lanthanum element was mainly distributed in the remineralized dentin. g–i) The TEM images with SAED pattern and elemental mapping show that the generated crystals in the DTs are La‐HAP with typical (002) and (211) planes. The SAED pattern was obtained from the point marked with a white pentagram in (h). j) Schematic diagram of the dentin permeability test device. k) The air pressure through the dentin disks was detected after different desensitizing treatments (PCCP mineralization media, Gluma desensitizer, Duraphat desensitizer, negative control) following abrasive, acidic, or ultrasonic challenges. The data are presented as mean ± SD, *n* = 3, and one‐way ANOVA testing followed by a Tukey post‐hoc test was carried out across groups. “*” indicates statistically significant difference (*p* < 0.05) and “ns” indicates no statistically significant difference (*p* > 0.05).

The key to the remineralization of DDM is the permeation of mineralization medium through dense DDM and subsequent remineralization of the DDM.^[^ ^57^, ^58^ ^]^ The PCCP process has been demonstrated to rapidly induce remineralization of DDM and in‐depth occlusion of DTs.^[^ ^27^, ^28^ ^]^ This might be attributed to the pH‐dependent electrostatic attraction between the electropositive PAsp‐Ca complexes along with free Ca^2+^ ions and the electronegative collagen fibrils of the DDM and the HAp inwall of DTs.^[^ ^27^ ^]^ Herein, the 4‐6‐µm thick DDM was fully remineralized (Figure 4) and the exposed DTs were compactly occluded to a depth of 100 µm with La‐HAp (**Figure** **5**) via PCCP process. Specifically, once the dentin disk was sequentially applied with PAsp‐Ca‐La suspension and phosphate solution each for 2 h, a large amount of amorphous minerals were generated throughout the whole demineralized layer (Figure S9, Supporting Information). Afterward, these amorphous minerals gradually transformed to La‐HAp within DDM for 7 d as evidenced by the TEM and HRTEM images with SAED pattern (Figure 4c–h).^[^ ^14^, ^52^, ^53^ ^]^ The elemental mapping results confirmed the incorporation of La^3+^ within the remineralized DDM (Figure 4i–k). The La/(Ca + La) ratio of crystals inside the remineralized layer was 0.11 ± 0.05 (Figure 4i–k), which was lower than La‐doped mineralization of single‐layer type I collagen fibrils with a La/(Ca + La) ratio of 0.23 ± 0.09 (*p* < 0.05, Figure S3c,d, Supporting Information). This is a challenge of La‐doped remineralization of 3D DDM.^[^ ^15^ ^]^ The ATR‐FTIR spectra indicate that the phosphate absorbance bands of remineralized dentin (1021, 600, and 560 cm^−1^) were shifted compared to those of native dentin (1029, 601, and 561 cm^−1^, Figure S10, Supporting Information). This might be attributed to the formation of La‐HAp within the remineralized layer because larger interplanar spacings (Figure 4h) resulted from the incorporation of La^3^⁺ into the HAp lattices.^[^ ^42^, ^45^ ^]^ Furthermore, the mechanical properties of heavily remineralized DDM (hardness modulus: 0.67 ± 0.05, elastic modulus: 18.32 ± 4.54 GPa) could be restored close to those of the intact dentin (hardness modulus: 0.83 ± 0.26, elastic modulus: 21.18 ± 6.11 GPa, *p* < 0.05, Figure 4l–n). In contrast, although the etched human dentin disks were incubated in daily‐refreshed artificial saliva for 7 d, no noteworthy remineralization was detected (Figure S11, Supporting Information) due to the lack of polyelectrolytes and insufficient mineral ions.^[^ ^15^ ^]^ Moreover, the PILP process is not a potent method for La‐doped remineralization of the DDM, as it only achieved a 500‐nm‐thick remineralization at the bottom of the DDM after 7 d (Figure S12, Supporting Information). This is consistent with the segmental intrafibrillar mineralization of reconstituted collagen fibrils (Figure S5, Supporting Information).

Meanwhile, the scanning electron microscopy (SEM) and TEM results revealed the achievement of the in‐depth occlusion of DTs via PCCP process (Figure 5a–i), while no occlusion of DT was obtained in the negative controls (Figure S13, Supporting Information). In the PCCP group, the DTs of dentin disks were completely covered and deeply occluded over 100 µm by compact La‐doped calcium phosphate crystals (Figure 5a–f). These crystals formed within DTs were identified by elemental mapping and SAED pattern as La‐HAp, with the La/(Ca + La) and (Ca + La)/P ratios of 0.12 ± 0.02 and 1.62 ± 0.09, respectively, and specific arcs of the (002) and (211) (Figure 5g–i).^[^ ^14^, ^52^, ^53^ ^]^ It has been reported that the wall of DTs is electronegative, and the electrical potentials escalate from the enamel side (−183 ± 21 mV) toward the pulp side (−280 ± 36 mV).^[^ ^11^ ^]^ Therefore, it is believed that the electropositive PAsp‐Ca‐La complexes (+6.05 ± 3.14 mV) could infiltrate into the deep region of the DTs via electrostatic attraction like PAsp‐Ca complexes.^[^ ^27^ ^]^ Subsequently, the complexes further attracted phosphate groups to form La‐ACP, which gradually transformed into stable La‐HAp, ultimately achieving the in‐depth occlusion of DTs (Figure 5a–i).

Nevertheless, in the PCCP group, the DTs remained mostly covered and deeply occluded even after the abrasive, acidic or ultrasonic challenges (Figure S14, Supporting Information). This might be attributed to the dense coverage and in‐depth occlusion with highly crystalline and acid‐resistant La‐HAp crystals.^[^ ^42^, ^43^, ^44^ ^]^ However, the SEM results in this study indicated that the DT occlusion effects of Gluma and Duraphat desensitizers were limited and could not withstand abrasive, acidic or ultrasonic challenges (Figures S15, S16, Supporting Information). Hence, the air pressure through the dentin disks after the treatment with PCCP mineralization media (156.70 ± 5.78 kPa) was significantly higher than that of the Gluma group (30.00 ± 2.00 kPa), Duraphat group (32.67 ± 6.43 kPa) or negative control group (25.00 ± 5.00 kPa, *p* < 0.05, Figure 5j,k). Although the permeabilities of the dentin disks treated with PCCP mineralization media were increased after the abrasive, acidic or ultrasonic challenges (*p* < 0.05), they remained significantly lower than those of the other three groups respectively (*p* < 0.05, Figure 5j,k). Therefore, the PCCP mineralization media show the superior application advantages in the DH management compared to the commercially available desensitizers.

**Figure 6:**
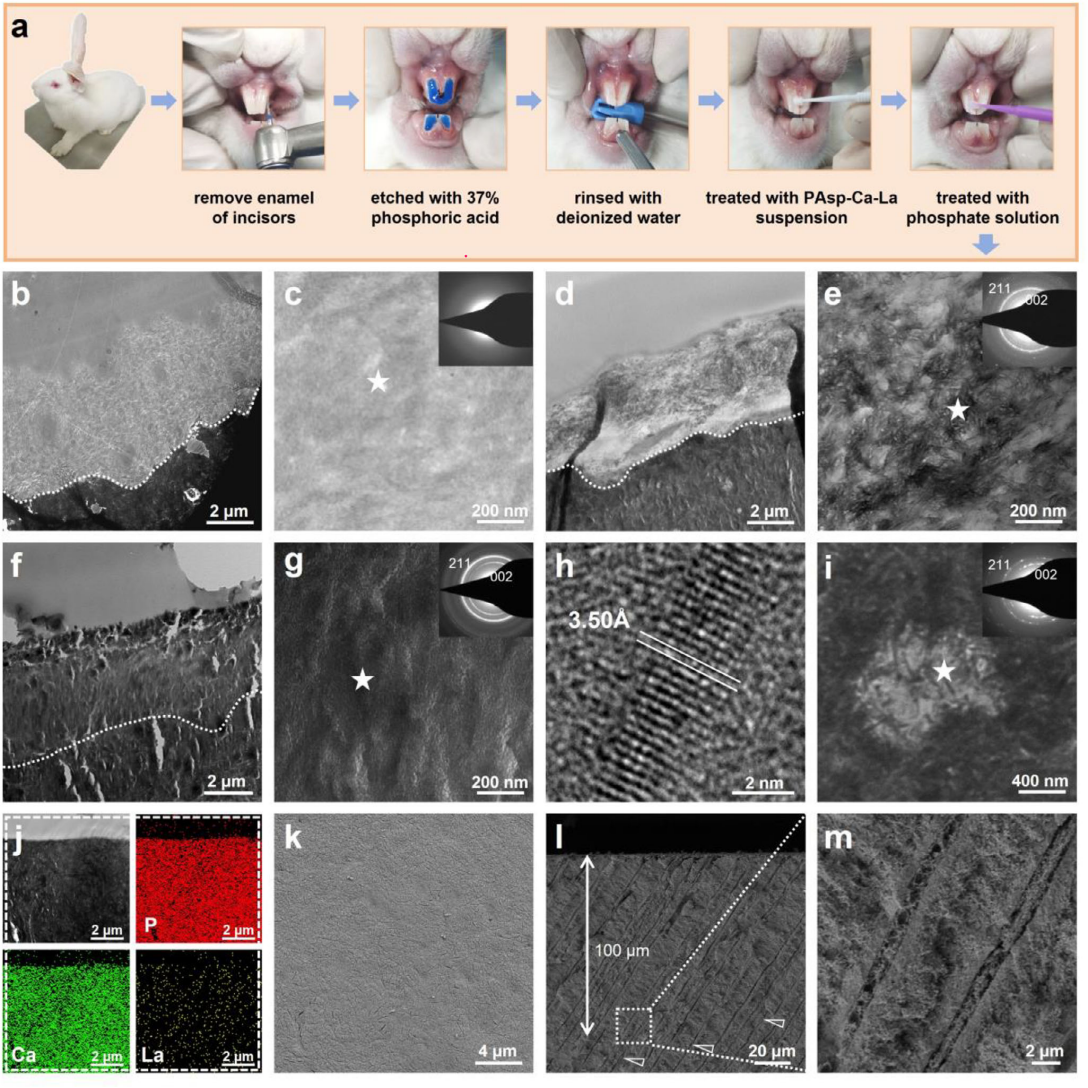
In vivo remineralization of DDM and occlusion of DTs via PCCP process. a) Schematic illustration of the in vivo experimental procedures of PCCP process. b,c) TEM images with SAED pattern indicate that the demineralized dentin could not be well recovered in the oral cavity of rabbits for 7 d. d,e) TEM images with SAED pattern of the demineralized dentin after the treatment of PCCP mineralization media and 1 d of incubation reveal a partial remineralization of the demineralized dentin with La‐HAp after the dentin treatment of PCCP mineralization media and 1 d of incubation. f–i) TEM and HRTEM images with SAED patterns show the completely remineralized dentin and in‐depth occluded DTs with dense La‐HAp crystals after 7 d of incubation. The SAED patterns were obtained from the points marked with white pentagrams in (c,e,g,i). j) The elemental mapping of the remineralized dentin indicates the abundant distributions of calcium, lanthanum, and phosphorus elements. k–m) SEM findings showed that the remineralized dentin surface was completely covered by dense crystals (k) and the DTs were compactly occluded by crystals over 100 µm by crystals (l,m). The white arrowheads in (l) represent the occluding minerals with DTs.

Furthermore, the in vivo efficacy of the PCCP process was evaluated using New Zealand rabbit incisor dentin (**Figure** **6**a). The DDM of the rabbit incisor without the intervention of PCCP mineralization media exhibited a poor recovery (Figure 6b,c), and collagen fibrils were naked even after being incubated in the oral environment for 7 d (Figure S17, Supporting Information). Moreover, the orifices of DTs were still uncovered and the DTs were hollow without identifiable crystals (Figure S17, Supporting Information). However, once the mineralization strategy via PCCP process was conducted on the dentin surface, the La‐doped remineralization of DDM and occlusion of DTs over 100 µm were accomplished as revealed by TEM and SEM results (Figure 6d–m). As identified by the HRTEM images with SAED pattern and elemental mapping (Figure 6g–j), these formed La‐HAp crystals in the remineralized dentin matrix and DTs might provide antibacterial properties for the recovered dentin tissues.^[^ ^45^, ^46^ ^]^

### 2.4 In Vitro and In Vivo Antibacterial Properties of the Remineralized Dentin

*S. Mutans* is one of the most widely recognized and extensively studied cariogenic bacteria due to its specific ability to form dental biofilms and produce organic acids, which contributes to rapid dentin demineralization.^[^ ^3^, ^59^, ^60^ ^]^ Moreover, *S. Mutans* could further invade the deep dentin micro‐structure through DTs, which is critical to the rapid progression of dentin caries.^[^ ^4^, ^5^, ^6^ ^]^ Hitherto, the appreciable antibacterial properties of La‐HAp have been extensively verified in the biomedical sciences.^[^ ^45^, ^46^ ^]^ In this study, we proposed a PCCP process for the remineralization of DDM and in‐depth occlusion of DTs with La‐HAp to obtain the antibacterial properties of dentin surface. Therefore, the in vitro and in vivo antibacterial experiments were carried out to evaluate the antibacterial properties of the remineralized dentin against *S. mutans*.

**Figure 7:**
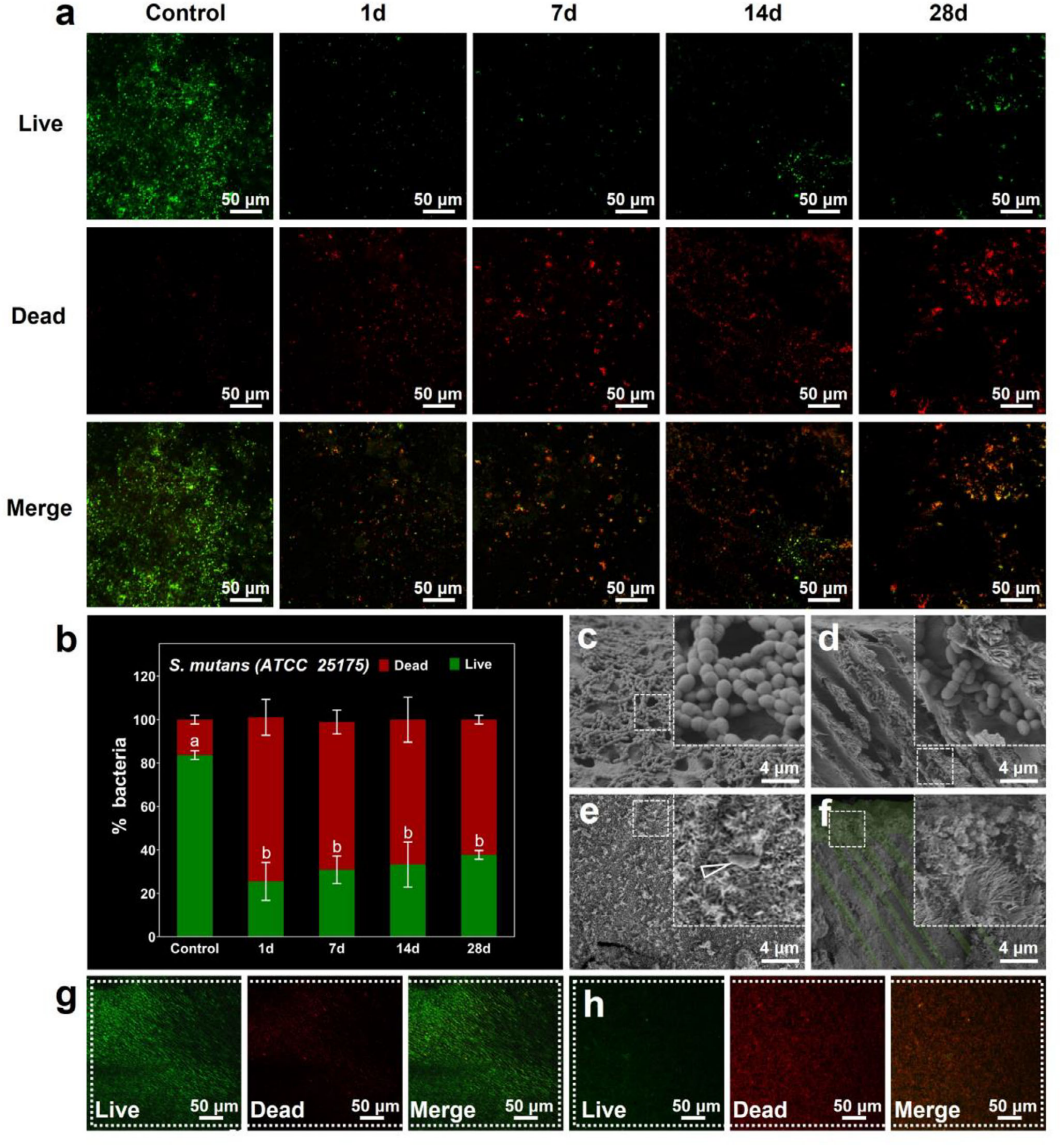
In vitro (a–f) and in vivo (g,h) antibacterial properties of the de‐ and remineralized dentin against *S. mutans*. a) CLSM images of the demineralized dentin disks (control) and remineralized dentin disks (1, 7, 14 and 28 d) after in vitro co‐cultivation with *S. mutans* suspension for 1 d. b) Bar chart shows the percentages of dead bacteria (red bars) and live bacteria (green bars) for each group in (a). The data are presented as mean ± SD, *n* = 5, and one‐way ANOVA testing followed by a Tukey post‐hoc test was carried out across groups. Different letters above the bars indicate significant differences (*p* < 0.05) in bacterial proportion between groups. c,d) SEM findings of the demineralized dentin disk showed that a lot of bacteria adhered to the dentin surface (c) and even invaded along the DTs (d) after in vitro co‐cultivation with *S. mutans* suspension for 1 d. e,f) SEM results of the completely remineralized dentin disks showed only a few shrunken bacteria (white arrowhead in (e)) on the dentin surface and the DTs were compactly occluded by platelet‐like crystals (the green‐stained area in (f)) with no bacteria presence after in vitro co‐cultivation with *S. mutans* suspension for 1 d. The La‐doped remineralized dentin layer maintained its integrity with no exposed collagen fibrils (the green‐stained area in (f)). g,h) CLSM images of the rabbit incisor dentin (g: demineralized dentin, h: remineralized dentin) after 7 d of incubation in the oral cavity followed by 1 d of co‐cultivation with *S. mutans* suspension.

In the in vitro antibacterial experiment, *S. mutans* were inoculated onto the demineralized dentin disks (control) and remineralized dentin disks as illustrated in representative confocal laser scanning microscopy (CLSM) images (**Figure** **7**a). The *S. mutans* that adhered to the demineralized dentin disks were diffusely distributed, with a relatively high density of live bacteria (Figure 7a). Contrarily, the densities of live bacteria on the remineralized dentin disks were dramatically reduced (Figure 7a), with the high percentages of dead bacteria being 75.58%, 68.07%, 66.73%, and 62.29%, respectively, after 1, 7, 14, and 28 d of incubation in artificial saliva and 1 d of co‐culture with *S. mutans*, compared to 16.22% in the demineralized dentin disks (*p* < 0.05, Figure 7b). Likewise, the SEM findings showed that numerous bacteria adhered on the surface of demineralized dentin disk (Figure 7c) and even deeply invaded along the DTs (Figure 7d). However, the surface of the remineralized dentin disk showed only a few bacteria with a collapsed morphology (Figure 7e), and the DTs were compactly occluded by platelet‐like crystals in absence of bacteria (Figure 7f). The La‐doped remineralized dentin layer maintained its integrity even when confronted with bacterial attack (Figure 7e,f). Both the CLSM and SEM results revealed the explicit antibacterial efficacy of the La‐doped remineralized dentin in inhibiting the bioactivity of *S.mutans* and blockading its invasion path. Furthermore, the in vivo antibacterial efficacy of the La‐doped remineralized dentin was confirmed in the incisor dentin of New Zealand rabbits. The bacteria on the demineralized dentin was predominantly live (percentage of live bacteria: 87.40%, Figure 7g), whereas the bacteria on remineralized dentin was primarily dead (percentage of dead bacteria: 70.38%, Figure 7h). Hence, both in vitro and in vivo, this La‐doped strategy effectively incorporated functional La^3+^ tightly into the remineralized dentin and extensively stored them within DTs, offering the potential for achieving long‐term antibacterial properties.^[^ ^45^, ^46^ ^]^

**Figure 8:**
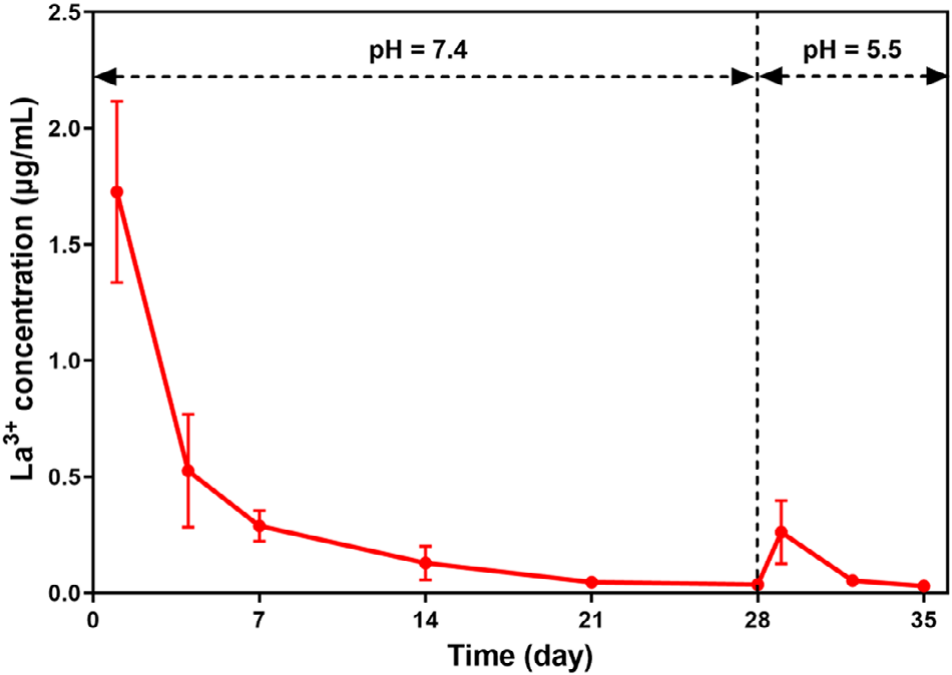
The release profiles of La^3+^ from the remineralized dentin disks after incubation in artificial saliva. After the demineralized dentin disks were treated with PCCP mineralization media, they were each immersed in 5 mL of artificial saliva (refreshed daily, pH 7.4) at 37 °C. The daily release of La^3+^ ions decreased over time, eventually reaching a slow‐release plateau phase after 21 d. When the pH value of artificial saliva was adjusted to 5.5 as critical demineralization pH value after 28 d of incubation, the release of La^3+^ ions immediately reached a peak. The data are presented as mean ± SD, *n* = 3.

This study attempted to construct a La‐doped remineralized dentin layer with compactly occluded DTs by La‐HAp as a pH‐dependent instantly responsive antibacterial strategy. The inductive coupled plasma mass spectrometer (ICP‐MS) result (**Figure** **8**) indicate that La^3+^ ions were slowly released from the remineralized dentin disks to the artificial saliva (pH 7.4), reaching a plateau at 21–28 d. Once the remineralized dentin disks were confronted with the critical pH of 5.5 to simulate the cariogenic bacteria‐directed dentin demineralization,^[^ ^3^ ^]^ a large amount of La^3+^ ions were immediately released once again (Figure 8), which was ascribed to the superior antibacterial properties of the remineralized dentin disk for 28 d (Figure 7). These data might indicate that the doped La^3+^ within the remineralized dentin and DTs are stored as stable La‐HAp crystal structure for responding to a secondary attack of *S. mutans*.^[^ ^45^, ^46^ ^]^

### 2.5 Biocompatibility Evaluation

**Figure 9:**
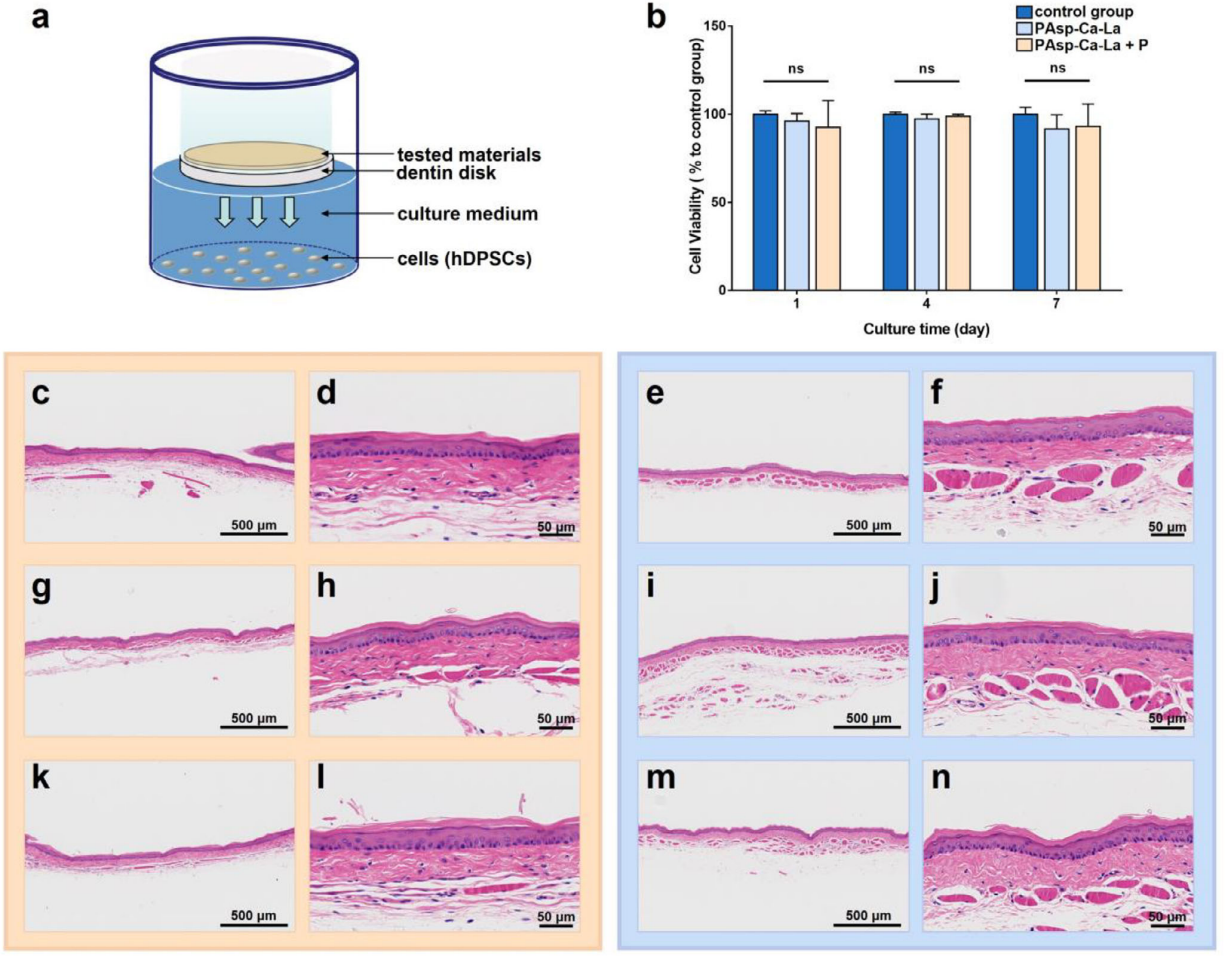
Cytotoxicity and oral mucosa irritation test. a) Schematic diagram of the TDD model to evaluate the cytotoxicity of the experimental materials to hDPSCs. b) Cell viability of hDPSCs after treatment of PAsp‐Ca‐La suspension with or without phosphate solution through the TDD model. The data are presented as mean ± SD, *n* = 3, and one‐way ANOVA testing followed by a Tukey post‐hoc test was carried out across groups. “*” indicates statistically significant difference (*p* < 0.05) and “ns” indicates no statistically significant difference (*p* > 0.05). c–n) Histological sections of gold hamsters’ oral mucosa that were treated with PAsp‐Ca‐La suspension (c,d), phosphate solution (g,h), and sequentially (k,l), as well as self‐control (e,f,i,j,m,n) treated with 0.9% NaCl. No leucocyte infiltration, edema of the mucosa, or vascular congestion were observed in all groups.

It is imperative to conduct an assessment of the biological safety of the PCCP mineralization media before the potential clinical applications. In this study, the biocompatibility of the PAsp‐Ca‐La suspension and phosphate solution was evaluated by assessing their cytotoxicity to human dental pulp stem cells (hDPSCs) using a transwell dentin disk (TDD) model (**Figure** **9**a) and standardized oral mucosa irritation test.^[^ ^61^ ^]^ No cytotoxicity (Figure 9b) or mucosal irritation (Figure 9c–n) was observed after separate or sequential treatment with the PAsp‐Ca‐La suspension and phosphate solution, nor was the control groups. Thus, the PCCP mineralization media demonstrate adequate biological safety for the dental pulp and direct contact with the oral mucosa.

## 3 Conclusion

In summary, the pioneering PCCP process, for the first time, achieved La‐doped intrafibrillar mineralization without any modification of collagen fibrils, inspiring the construction of an antibacterial remineralized dentin surface and in‐depth occlusion of DTs over 100 µm with La‐HAp both in vitro and in vivo. This multifunctional strategy could endow the remineralized dentin surface with antibacterial ability to kill off the cariogenic bacteria (*S. mutans*) and to block bacterial invasion path in progression of the caries, which might be a promising approach for the management of dentin caries, particularly when accompanied with dentine hypersensitivity.

## 4 Experimental Section

### Chemicals and Materials

All the materials and chemical reagents for this study were obtained from commercial sources. They included calcium chloride dihydrate (CaCl_2_·2H_2_O), lanthanum(III) chloride heptahydrate (LaCl_3_·7H_2_O), potassium phosphate (K_2_HPO_4_, Aladdin, China); sodium hydroxide (NaOH), chloramine T trihydrate and glutaraldehyde solution (25 wt.%, Sinopharm Chemical Reagent Co., China); potassium chloride (KCl), absolute ethanol (Macklin, China); 4‐(2‐hydroxyethyl)‐1‐piperazineethanesulfonic acid (HEPES) and hydrochloric acid (HCl); phosphoric acid (85 wt.%, Sigma–Aldrich, USA); Pure rat tail type I collagen (Gibco‐Invitrogen, USA); poly‐L‐asparate acid (PAsp, Mw: 7000–8000 Da, Aike‐Reagent, China); phosphate buffer saline (PBS) and Brain Heart Infusion (BHI, Beijing Solarbio, China).

### Preparation of PCCP Mineralization Media and Artificial Saliva

To achieve an atomic ratio of La/(Ca + La) at 0.1, a total of 11.921 g CaCl_2_·2H_2_O and 3.346 g LaCL_3_·7H_2_O was concurrently dissolved into 15 mL of deionized water. Afterward, 1.325 mL of 100 g L^−1^ PAsp solution was added and sufficiently stirred to form a PAsp‐Ca‐La suspension. The pH was standardized to 9.5 using a 10 м NaOH solution. The PAsp‐Ca‐La suspension reached a final concentration of 5 g L^−1^‐3.06 м‐0.34 м, referring to 5 g L^−1^ PAsp, 3.06 м Ca^2+^ and 0.34 м La^3+^. To achieve an atomic ratio of (Ca + La)/P at 1.67, a 2.04 м K_2_HPO_4_ solution served as the phosphate solution, with its pH value also adjusted to 9.5 using HCl solution (1 м). The artificial saliva (0.9 mм KH_2_PO_4_, 1.5 mм CaCl_2_, 20 mм HEPES, 1.0 mм NaN_3_, 130 mм KCl, pH 7.4) was prepared and filtrated using a 0.22‐µm filter (Millipore, USA).^[^ ^27^ ^]^

### Characterization of PAsp‐Ca‐La Complexes

An aliquot (3 µL) of PAsp‐Ca‐La suspension was dripped onto a carbon‐and‐formvar‐coated nickel grid, kept on parafilm for 20 min, dehydrated with ethanol, and analyzed by field emission scanning electron microscopy (FESEM, Nova Nano 450, Thermo FEI, Czech) equipped with energy‐dispersive X‐ray analysis (EDX), and HRTEM (FEI Tecnai G2 F20 STWIN, FEI, USA) equipped with SAED. Zeta potential measurements of the PAsp‐Ca‐La suspension were conducted in triplicate by Malvern Instrument Zetasizer (Malvern Panalytical, UK). Sizes of the nanoparticles within the PAsp‐Ca‐La suspension were determined in triplicate by DLS after a ten‐fold dilution.

### Preparation and Characterization of PAsp‐Stabilized ACP and La‐ACP Solution

PAsp‐stabilized ACP solution (9.5 mм Na_2_HPO_4_, 1.67 mм CaCl_2_, 240 µg mL^−1^ PAsp and 150 mм NaCl) and PAsp‐stabilized La‐ACP solution (9.5 mм Na_2_HPO_4_, 1.503 mм CaCl_2_, 0.167 mм LaCl_3_, 240 µg mL^−1^ PAsp and 150 mм NaCl) were prepared using previously described methods with slight modifications.^[^ ^18^ ^]^ The analysis of ACP and La‐ACP nanoparticles was conducted as mentioned above.

### Characterization of Mineralized Reconstituted Type I Collagen Fibrils

The reconstitution of single‐layer type I collagen fibrils on the grid was conducted as detailed in the Supporting Information.^[^ ^62^, ^63^ ^]^ To verify the assessment of collagen self‐assembly, the grid was stained with 10 µL of 0.5 wt.% uranium acetate solution and then observed by TEM (JEM‐1400 Flash, JEOL, Tokyo, Japan, Figure S18, Supporting Information). The collagen‐coated grids were successively floated upside‐down over 100 µL of PAsp‐Ca‐La suspension and 100 µL of phosphate solution each for 1 h, followed by 5‐min blot‐drying. Afterward, each grid was floated on 2 mL of artificial saliva, which was refreshed daily, for 1 and 4 d. For comparative analyses, collagen‐coated grids were treated following the same procedure except that a Ca‐La suspension (3.06 м–0.34 м, pH 9.5) was utilized instead of the PAsp‐Ca‐La suspension. Another set of collagen‐coated grids were floated on the PAsp‐stabilized La‐ACP solution for 4 d, with the solution being refreshed every 12 h. All the retrieved grids were rinsed and dehydrated with ethanol for TEM, FESEM with EDX, HRTEM with SAED analyses. Additionally, the mineralized collagen gels were obtained as detailed in the Supporting Information and then analyzed with ATR‐FTIR (Nicolet iS10, Thermo Scientific, USA).

### Establishment of Dentin Demineralization and Hypersensitivity Model

The caries‐free human third molars were collected with the approval of the Medical Ethics Committee of Stomatology Hospital, Zhejiang University School of Medicine (No. 2024‐079) and informed consents were obtained from all donors. After extraction, the teeth were maintained at 4 °C in a 0.5% chloramine T solution and utilized within one month. Under water‐spraying, 1‐mm‐thick dentin disks were perpendicularly sectioned from the mid‐coronal areas with a slow‐speed diamond saw (Buehler, IL, USA), followed by sequential polishing using 600‐, 1200‐, and 2400‐grit SiC paper. After 15 s of etching with 37% phosphoric acid and 60 s of rinsing with deionized water, a DDM layer of 4–6 µm thickness and extensively exposed DTs were generated.^[^ ^64^ ^]^

### In Vitro Remineralization of DDM

The demineralized dentin disks were successively applied with PAsp‐Ca‐La suspension and phosphate solution each for 2 h, followed by blot‐drying for 5 min. Subsequently, each dentin disk was placed in 5 mL of artificial saliva (refreshed daily) for periods of 1 and 7 d. Demineralized dentin disks with or without further incubation in artificial saliva were employed as controls. For comparison, demineralized dentin disks were placed in the PAsp‐stabilized La‐ACP solution, which was refreshed at 12‐h intervals for 7 d. All the dentin disks were retrieved and subjected to a progressive dehydration process using ethanol concentrations ranging from 30% to 100%. All the specimens were treated with acetone, encased into epoxy resin, and transversely sectioned into 90‐nm‐thick slices for the analyses of TEM, FESEM with EDX, HRTEM with SAED analyses.

### ATR‐FTIR Spectra of Dentin

The native and demineralized dentin surfaces as well as the remineralized dentin surfaces treated with PCCP mineralization media as above‐mentioned were air‐dried and analyzed using ATR‐FTIR. The spectra were acquired over a wavenumber range of 400–4000 cm^−1^, with a resolution of 4 cm^−1^ and 40 scans.

### Nanoindentation Testing of Dentin

The nanomechanical properties (hardness and elastic modulus) of the native, demineralized and remineralized dentin disks were analyzed by Nanoindenter G200 (Agilent Technologies, USA) and calculated using the loading and unloading force‐displacement curve. Five points for each specimen were indented for 2 s with a peak force of 10 mN. Three specimens were evaluated for each group.

### Release Profiles of La^3+^

Following the sequential application of PAsp‐Ca‐La suspension and phosphate solution to the demineralized dentin disks (5 × 5 × 1 mm, *n* = 3), each dentin disk was placed in 5 mL of artificial saliva (refreshed daily, pH 7.4). From day 29, the pH value of the artificial saliva was adjusted to 5.5 to simulate the cariogenic bacteria‐directed dentin demineralization.^[^ ^3^ ^]^ The concentrations of released La^3+^ into the artificial saliva were measured on days 1, 4, 7, 14, 21, 28, 29, 32, and 35. At each checkpoint time, the retrieved artificial saliva, which did not contain the dentin disk, was ten‐fold diluted with HCl solution (1 м) and then measured by ICP‐MS 7800 (Agilent Technologies, US).

### In Vitro Occlusion of DTs

In the PCCP group, the etched 1‐mm‐thick dentin disks were sequentially treated with PAsp‐Ca‐La suspension and phosphate solution, as mentioned above. For comparison, Duraphat desensitizer (Colgate‐Palmolive, UK) and Gluma desensitizer (Kulzer, Germany) were applied following the previously reported procedures and served as positive controls, while the etched dentin disks served as negative controls.^[^ ^65^, ^66^ ^]^ All the treated specimens (*n* = 24 per group) were each placed in 5 mL of artificial saliva (refreshed daily) for 7 d. In each group, six specimens received no further treatment, six were brushed with toothpaste for 1 min, six were subjected to a 1‐min treatment with 6 wt.% citric,^[^ ^67^ ^]^ and six were subjected to a 10‐min ultrasonic treatment. All the dentin disks were then retrieved and subjected to a progressive dehydration process by ethanol solutions (30–100%). Subsequently, three specimens from each subgroup were split into two. Both the longitudinal and transverse surfaces of the specimen were gold‐sputtered and analyzed by SEM (Zeiss Gemini SEM 300, Oberkochen, Germany) with EDX. The other three specimens from each subgroup underwent the dentin permeability measurement using a modified dentin permeability test device following Li's work (Figure 5j).^[^ ^6^ ^]^

### In Vivo Remineralization of DDM and Occlusion of DTs

All the animal experiments in this study were approved by the Laboratory Animal Welfare and Ethics Committee of Zhejiang University (No. ZJU20240712) and were strictly conducted following the National Institutes of Health guide for the care and use of laboratory animals. Six New Zealand white rabbits (male, 2.3–2.5 kg, 12 weeks old, *n* = 3 per group) were anesthetized via intravenous injection with sodium pentobarbital at a dosage of 45 mg kg^−1^. A high‐speed turbine handpiece was used to remove the labial enamels of incisors for dentin exposure. After 15 s of etching with 37% phosphoric acid and rinsing with deionized water, the etched maxillary incisors were sequentially applied with PAsp‐Ca‐La suspension and phosphate solution as detailed above, while the mandibular incisors without any treatment were employed as controls. After 1 and 7 d, the rabbits were humanely euthanized, and their incisors were extracted for the TEM, FESEM with EDX, HRTEM with SAED analyses (right incisors), and SEM with EDX (left incisors) analyses.

### Preparation of Bacterial Suspension

The antibacterial properties of the remineralized dentin surface against *S. mutans* (ATCC 25175) was evaluated both in vitro and in vivo. A single *S. mutans* colony was inoculated into 5 mL of BHI broth and incubated under anaerobic conditions (10% H_2_, 80% N_2_, 10% CO_2_) at 37 °C overnight. Subsequently, the overnight culture was diluted 1:10 with fresh BHI broth and incubated until the optical density (OD) at 600 nm reached ≈0.5, as measured using a microplate reader (BioTek, USA).^[^ ^68^, ^69^ ^]^ Finally, the bacterial suspension was diluted to 10^8^ CFU mL^−1^ with fresh BHI broth containing 1% sucrose for further usage.^[^ ^69^ ^]^

### In Vitro Antibacterial Properties of Remineralized Dentin

Forty demineralized dentin disks (5 × 5 × 1 mm, *n* = 10 per group) were sequentially treated with PAsp‐Ca‐La suspension and phosphate solution, and then incubated in artificial saliva (refreshed daily) for 1, 7, 14, and 28 d. Ten demineralized dentin disks without treatment were designated as the control group. After sterilization by ultraviolet light overnight, the dentin disks were placed into 24‐well plate with the dentin‐treated surfaces upward. A 2‐mL volume of *S. mutans* suspension (10^8^ CFU mL^−1^) were inoculated into each well. Following 24 h of incubation under anaerobic condition at 37 °C, five dentin disks from each group were rinsed thrice by 1 mL of sterile PBS. Afterward, the Live/Dead bacterial viability kit (Invitrogen, USA) was used to visualize the adherent bacteria. Live bacteria fluoresced green when stained with Syto9, while membrane‐damaged bacteria fluoresced red when stained with propidium iodide (PI), as observed by CLSM (LSM800, Zeiss, Germany). Five images were randomly captured and analyzed to quantify the percentages of live and dead bacteria using Image J (NIH, Bethesda, MD).^[^ ^59^ ^]^ Another five dentin disks from each group were immersed in the glutaraldehyde (2.5%) at 4 °C for 24 h. After rinsing thrice with 1 mL of sterile PBS, the specimens were processed for SEM analysis.

### In Vivo Antibacterial Properties of Remineralized Dentin

Five New Zealand white rabbits were utilized and underwent the sequential treatment with PAsp‐Ca‐La suspension and phosphate solution following the procedures described above. After 7 d of incubation in the oral cavity of the rabbit, the maxillary incisors and mandibular incisors were each inoculated with 100 ul of *S. mutans* suspension (10^8^ CFU mL^−1^). Thereafter, the rabbits were provided with a soft diet supplemented with 2% sucrose. After 1 d, the rabbits were humanely euthanized, and their incisors were extracted and stained for CLSM analysis.

### Cytotoxicity Test

Cytotoxicity of the PAsp‐Ca‐La suspension (5 g L^−1^‐3.06 м‐0.34 м) and phosphate solution (2.04 м) was determined by a modified TDD model.^[^ ^61^ ^]^ Briefly, the transwell's insert membrane was substituted with an etched dentin disk (diameter = 5 mm, thickness = 1 mm) and secured in place with dental wax.^[^ ^61^ ^]^ After 12 h of sterilization by ultraviolet light, the dentin transwells were placed into a 24‐well plate, with each well filled with 500 µL of hDPSC suspension (5 × 10^4^ cells per well). The hDPSCs were provided by the institutional lab.^[^ ^70^ ^]^ The dentin surfaces were applied with PAsp‐Ca‐La suspension for 2 h with or without subsequent application with phosphate solution for another 2 h. The demineralized dentin disks applied with PBS served as negative controls. The proliferation rates of cells after 1, 4, and 7 d of incubation in the culture medium (*n* = 3 per group per checkpoint time) was analyzed by the Cell Counting Kit‐8 (Invigentech, USA). The OD was measured at 450 nm using a microplate reader. The relative cell viability (%) was ascertained by the comparison of the OD values with those of the negative controls.

### Oral Mucosa Irritation Test

A standard oral mucosa irritation test was performed according to ISO10993‐10:2010 guidelines. Nine golden hamsters (male, 110–130 g, *n* = 3 per group) were sourced from Beijing Weitong Lihua (Beijing, China). After one week of acclimatization, hamsters were anesthetized via intraperitoneal injection with sodium pentobarbital at a dosage of 60 mg kg^−1^. A cotton pellet (5 mm in diameter) was soaked in 50 µL of PAsp‐Ca‐La suspension or phosphate solution and then positioned into the hamster's left cheek pouch for 2 h, separately or sequentially. Simultaneously, a 0.9% NaCl‐soaked cotton ball was positioned in the right cheek pouch and served as a self‐control. Then the cotton balls soaked with the test materials were removed, and the cheek pouches were rinsed with 0.9% NaCl. After 1 d, the appearances of the cheek pouches were evaluated according to the grade of erythema grade (Table S1, Supporting Information). Subsequently, the hamsters were euthanized and the cheek pouch mucosae were harvested, followed by fixation in 4% paraformaldehyde for 24 h. After embedding in paraffin, the sections were processed by hematoxylineosin (HE) staining and subsequently evaluated using a light microscope. The oral mucosal irritation was assessed and graded by the criteria outlined in Table S2 (Supporting Information).

### Statistical Analysis

The SPSS software (version 26, Chicago, IL, USA) was utilized for statistical analysis, while the GraphPad Prism 8 software (San Diego, CA, USA) was employed for data visualization. All data were presented as mean ± standard deviation (mean ± SD). After confirming the normal distribution via the Shapiro‐Wilk test, the data of modulus, hardness, dentin permeability, cell viability, and bactericidal ratio were assessed by one‐way analysis of variance (ANOVA) test with Tukey's test. The threshold for statistical significance was set at the level of 0.05.

## Conflict of Interest

B.F., Z.Z., Z.W., and Y.X. declare that they obtained patents concerning the PCCP mineralization medium and its applications in China (No. ZL202210100316.2) and are applying the same patent in the United States (No. US20220031575A1). The other authors declare no conflicts of interest in this work.

## Supporting information



Supporting Information

## Data Availability

The data that support the findings of this study are available from the corresponding author upon reasonable request.
