# Supplementary material for: Polyaspartic Acid‐Calcium‐Lanthanum Complexes Induce Antibacterial Remineralization of Dentin and In‐Depth Occlusion of Dentinal Tubules
Source: Adv Sci (Weinh). 2025 Apr 27;12(25):2501340. doi: 10.1002/advs.202501340 (PMC12224999; doi:10.1002/advs.202501340)
Supplement: Supplementary file 1 — Supporting Information [file ADVS-12-2501340-s001.docx]

Supporting Information

**Polyaspartic Acid-Calcium-Lanthanum Complexes Induce Antibacterial Remineralization of Dentin and In-depth Occlusion of Dentinal Tubules**

*Ling Zhu^#^, Wei Liu^#^_,_ Yizhou Zhang, Zhifang Wu, Yiru Wang, Yuedan Xu, Haiyan Zheng, Hongli Zhang, Mengfei Yu, Xiaoting Jin, Zhe Wang^*^, Zihuai Zhou^*^ and Baiping Fu^*^*

L. Zhu

Department of Stomatology, Children’s Hospital, Zhejiang University School of Medicine, National Clinical Research Center for Child Health

Hangzhou, Zhejiang 310000, China

L. Zhu, W. Liu_,_ Y. Zhang, Z. Wu, Y. Wang, Y. Xu, H. Zheng, H. Zhang, M. Yu, X. Jin, Z. Wang, Z. Zhou and B. Fu

Stomatology Hospital, School of Stomatology, Zhejiang University School of Medicine,

Zhejiang Provincial Clinical Research Center for Oral Diseases,

Key Laboratory of Oral Biomedical Research of Zhejiang Province,

Engineering Research Center of Oral Biomaterials and Devices of Zhejiang Province,

Cancer Center of Zhejiang University

Hangzhou, Zhejiang 310000, China

^#^ Authors contributed equally.

^*^ **Corresponding author:** Baiping Fu [(fbp@zju.edu.cn)](mailto:(fbp@zju.edu.cn)).

^*^ Co-Corresponding author: Zihuai Zhou (11818406@zju.edu.cn)

^*^ Co-Corresponding author: Zhe Wang ([7319012@zju.edu.cn](mailto:7319012@zju.edu.cn))

**Keywords:** dental caries; intrafibrillar mineralization; polyaspartic acid-calcium-lanthanum complexes; antibacterial; dentinal tubule occlusion

**1. Additional Figures**


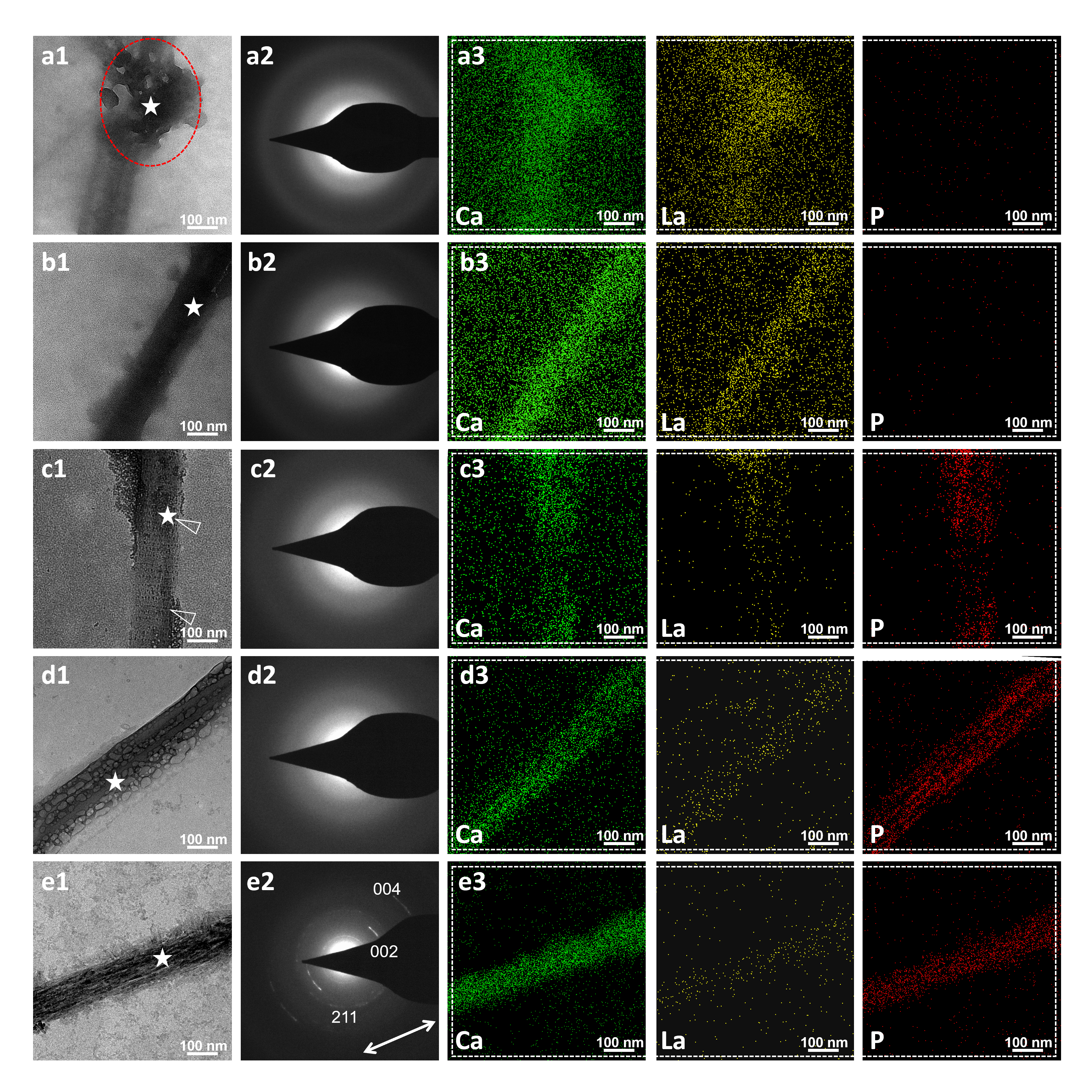


**Figure S1. HRTEM images with SAED patterns and elemental mapping of the collagen fibrils in PCCP process.** a) After treatment with PAsp-Ca-La suspension for 10 min, the PAsp-Ca-La complexes (red circle) were adsorbed onto collagen fibril (a1) with no distinct arc in the SAED pattern (a2), as highly-aggregated sites of calcium and lanthanum elements (a3). Concurrently, a substantial quantity of free Ca^2+^ and La^3+^ ions also infiltrated into the collagen fibril. b) After 1 h of treatment, the electron density of collagen fibril was further increased (b1), and the distribution of calcium and lanthanum elements became more uniform along the collagen fibril (b3), as the PAsp-Ca-La complexes, Ca²⁺ and La³⁺ ions adequately infiltrated into the collagen fibril. c) After subsequent treatment with phosphate solution for 10 min, some free Ca^2+^ and La^3+^ ions flowed out of the collagen fibril, leading to an uneven distribution of calcium and lanthanum elements within the collagen fibril. Meanwhile, phosphorus elements were preferentially accumulated in regions rich in calcium and lanthanum elements (white arrowheads in “c1”). d) After 1 h of treatment with phosphate solution, more phosphate groups entered the collagen fibrils and formed La-ACP throughout the collagen fibrils as confirmed by the SAED pattern (d2) and elemental mapping (d3). e) After incubation in artificial saliva for 4 d, the collagen fibrils were heavily mineralized with a uniform distribution of calcium, lanthanum and phosphorus elements along the collagen fibril. The c-axis of the crystals generated on collagen fibrils is almost parallel to the long axis of the collagen fibril (e1) with distinct arcs of the (002), (211) and (004). The white bidirectional arrow in “e2” represents the long axis of collagen fibril in “e1”. The SAED patterns (a2, b2, c2, d2, e2) were obtained from the points marked with white pentagrams (a1, b1, c1, d1,e1).


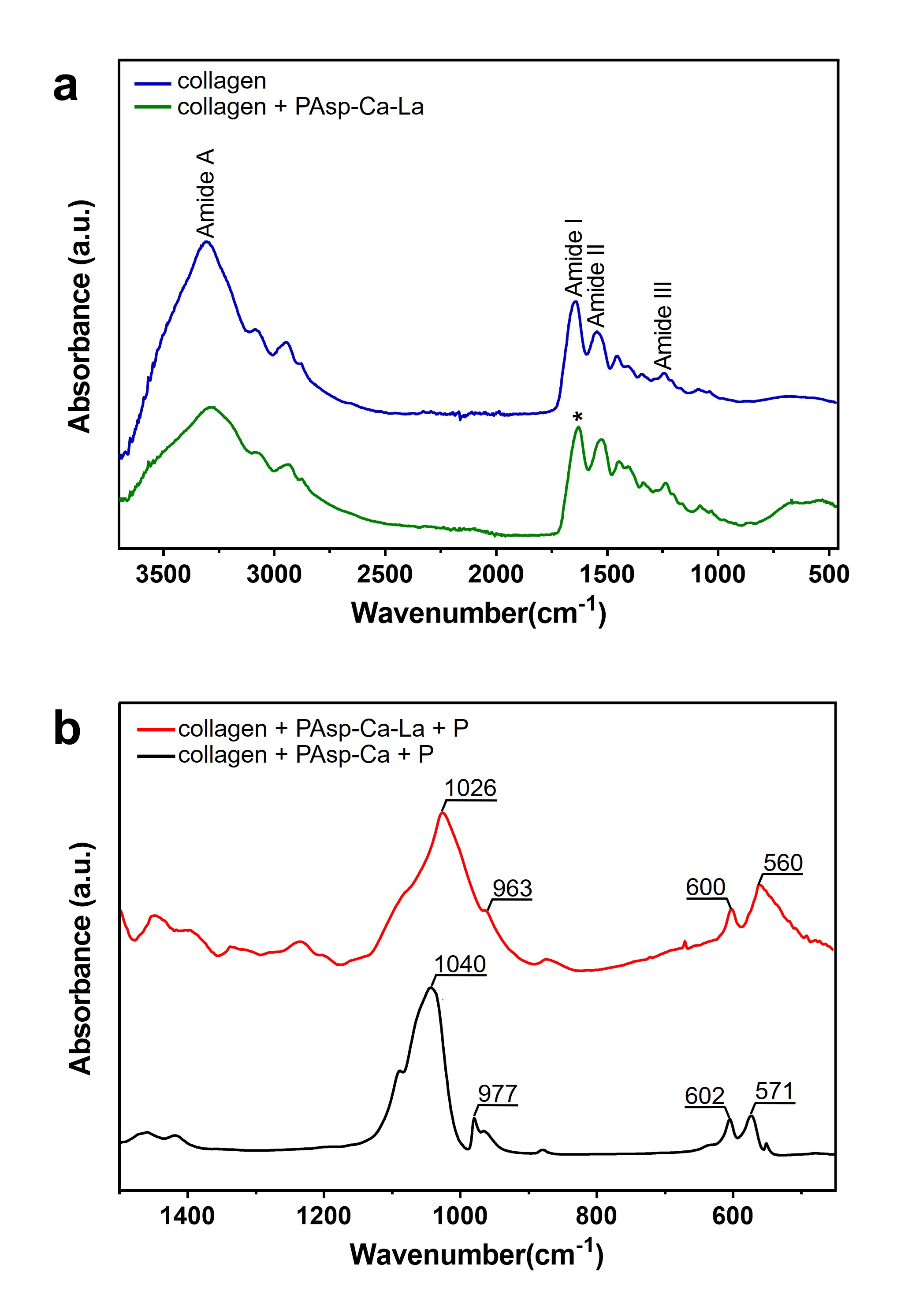


**Figure S2.** **ATR-FTIR** **spectra of the pure and treated collagen gels.** a) ATR-FTIR spectrum of the pure collagen gel shows the typical amide bands of collagen fibril, including C=O stretching of amide I (1633 cm^-1^), NH bending coupled with CN stretching of amide II (1541 cm^-1^), NH bending coupled with CN stretching of amide III (1236 cm^-1^) and NH stretching of amide A bands (3305 cm^-1^) respectively. After the collagen gel was treated with PAsp-Ca-La suspension, the peak of amide I band at 1633 cm^-1^ shifted to 1629 cm^-1^ (“*” in “a”). b) ATR-FTIR spectrum of the mineralized collagen gels treated with PAsp-Ca suspension, phosphate solution and incubated for 4 d, shows the characteristic absorbance bands of phosphate groups consistent with those of HAp. When the PAsp-Ca-La suspension was applied instead of PAsp-Ca suspension, the absorbance bands of phosphate groups shifts from 1040, 977, 602 and 571 cm^-1^ to 1026, 963, 600 and 560 cm^-1^, respectively.


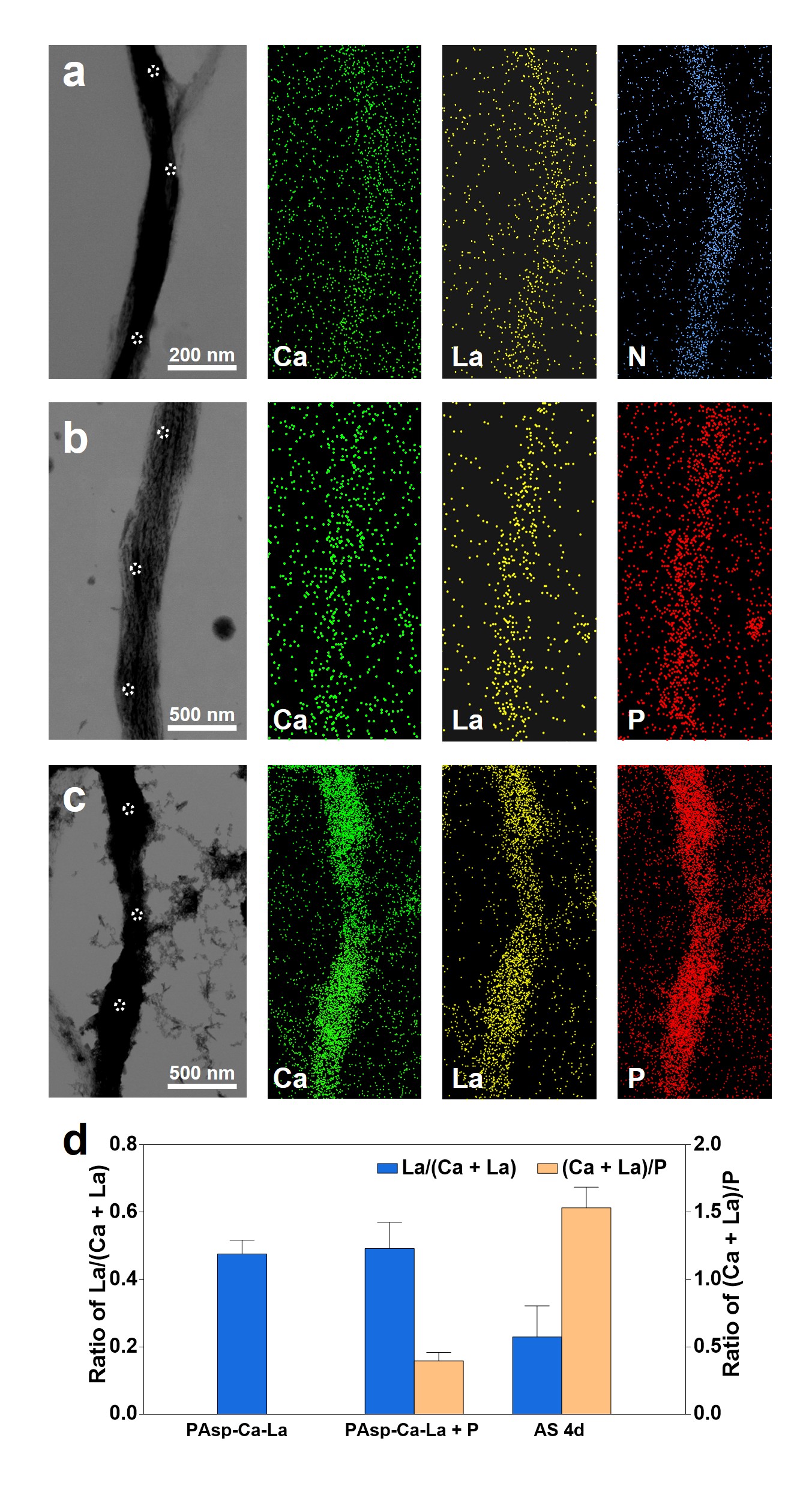


**Figure S****3.** **STEM images with elemental mapping of the treated reconstituted** **single-layer type I collagen fibrils via PCCP process.** a-c) STEM images with elemental mapping of the collagen fibrils treated with PAsp-Ca-La suspension for 1 h (a) followed by phosphate solution for 1 h (b) and subsequently incubated in artificial saliva for 4 d (c). Elemental mapping results indicated the distribution of calcium, lanthanum, nitrogen and phosphorus elements. d) The average La/(Ca + La) and (Ca + La)/P ratios of collagen fibrils at the three points (white dotted circles in “a-c”) are presented as mean ± SD.


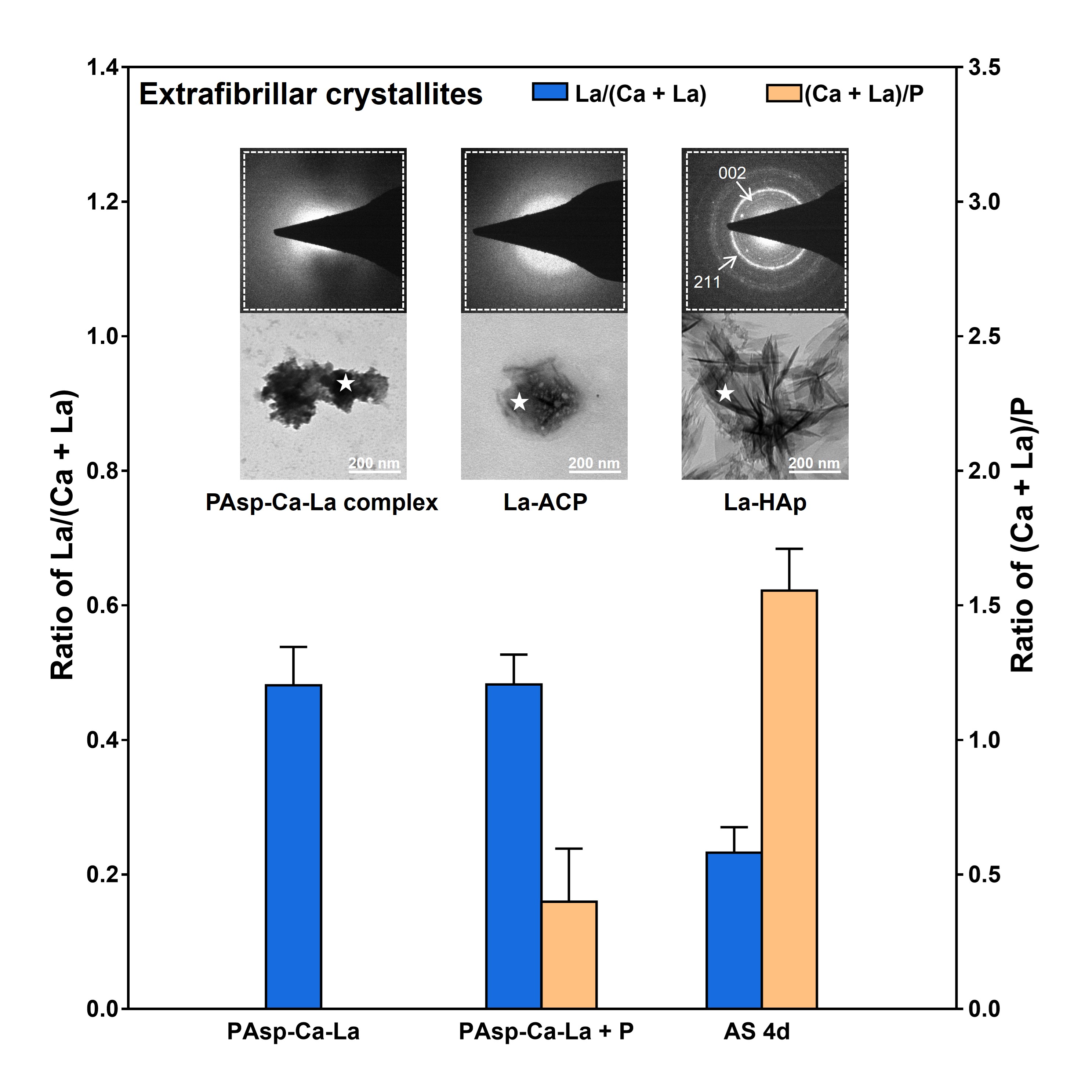


**Figure S4.** **HRTEM images with SAED patterns and EDX results of the** **extrafibrillar La-HAp via PCCP process.** The La/(Ca + La) and (Ca + La)/P ratios of minerals during crystallization process are presented as mean ± SD, n = 3. After the application of PAsp-Ca-La suspension for 1 h, the HRTEM image with SAED pattern displays the amorphous PAsp-Ca-La complexes with the La/(Ca + La) ratio of 0.48 ± 0.06. Subsequent treatment with phosphate solution for 1 h resulted in the formation of the amorphous La-ACP with the La/(Ca + La) and (Ca + La)/P ratios of 0.48 ± 0.04 and 0.40±0.20, respectively. After 4 d of incubation in artificial saliva, the amorphous La-ACP transformed into needle-like La-HAp with typical (002) and (211) planes. The La/(Ca + La) ratio of La-HAp decreased to 0.23 ± 0.04 and its (Ca + La)/P ratio increased to 1.56 ± 0.16. The SAED patterns were obtained from the points marked white pentagrams.


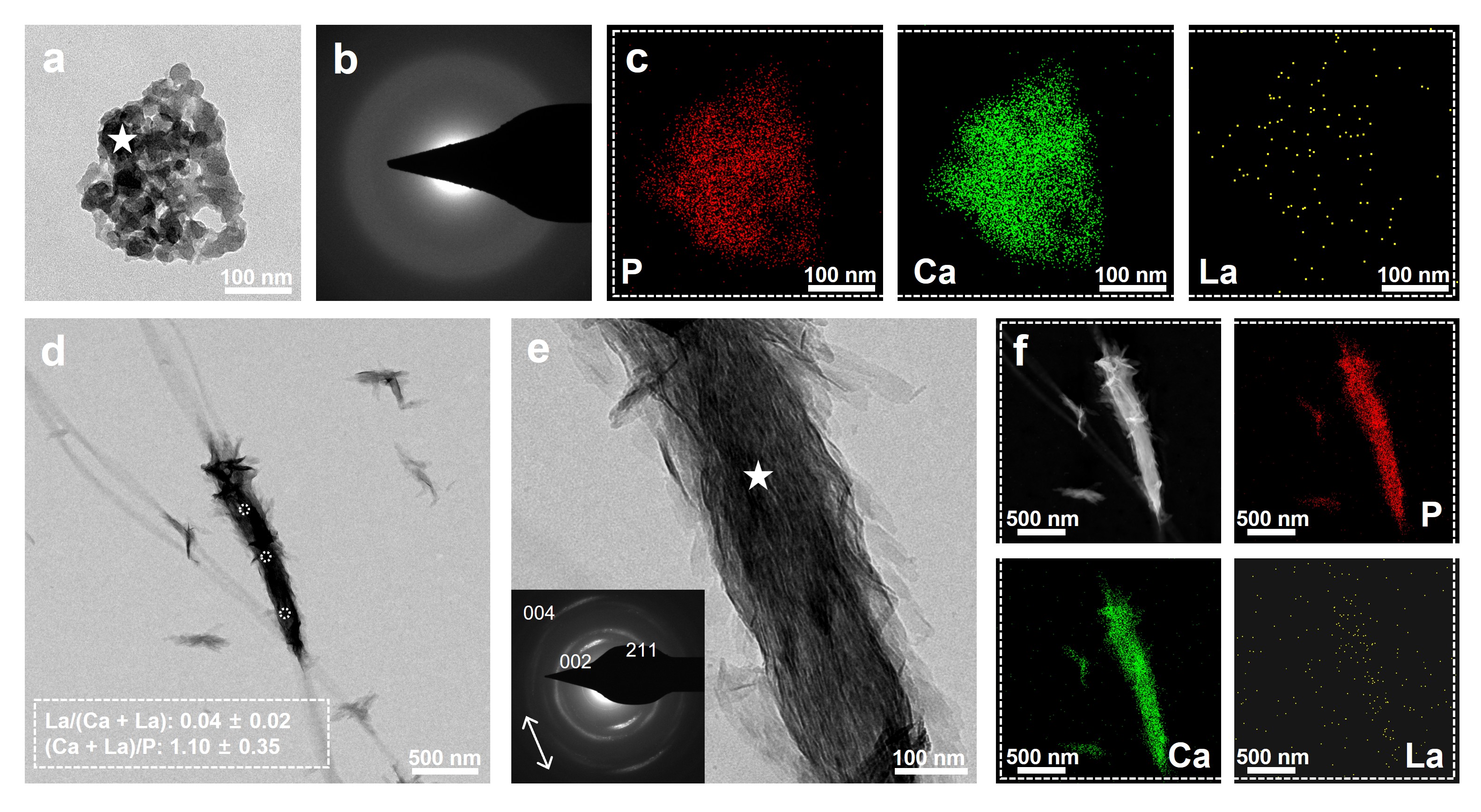


**Figure S5. TEM and HRTEM images with** **SAED patterns and elemental mapping of the La-ACP nanoparticles and the La-doped mineralization of collagen fibrils via** **PILP process.** a-c) HRTEM image with SAED pattern (obtained from the point marked with a white pentagram in “a”) and elemental mapping show an aggregated morphology of La-ACP nanoparticles as an amorphous entity containing phosphorus, calcium and lanthanum elements. The ratios of La/(Ca + La) and (Ca + La)/P were 0.03 and 1.02, respectively. d) TEM image with EDX data of the collagen fibrils treated with PAsp-stabilized La-ACP solution for 4 d shows that the segmental mineralization of collagen fibrils was achieved with the ratios of La/(Ca + La) and (Ca + La)/P as 0.04 ± 0.02 and 1.10 ± 0.35 respectively (mean ± SD of three white dotted circles areas). e) HRTEM image with SAED pattern (obtained from the point marked with a white pentagram in “d”) shows that the collagen fibril was heavily mineralized with the needle-like crystals with distinct (002), (211) and (004) planes. The c-axis of the crystals on the collagen fibrils is almost in parallel with the long axis of the collagen fibril (white bidirectional arrow). f) The elemental mapping indicates the distribution of the phosphorus, calcium and lanthanum elements along the collagen fibrils.


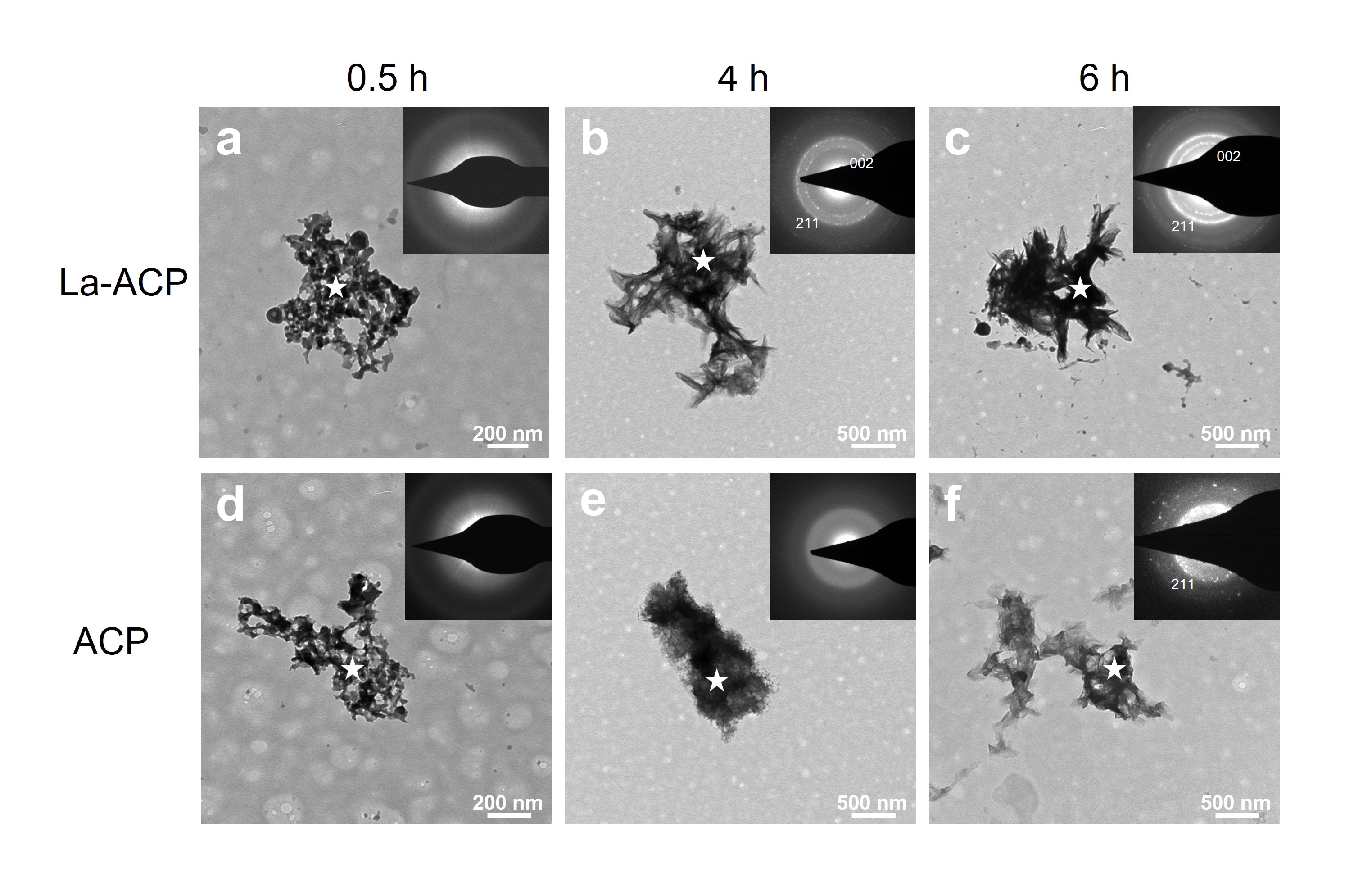


**Figure S6.** **HRTEM images with SAED patterns of the nanoparticles** **in PAsp-stabilized ACP and La-ACP solution.** a-c) The HRTEM images with SAED patterns show the amorphous La-ACP transformed into La-HAp crystals with typical (002) and (211) planes after 4 h. d-f) The HRTEM images with SAED patterns show the amorphous ACP transformed into HAp crystals with weak (211) plane after 6 h. The SAED patterns were obtained from the points marked with white pentagrams.


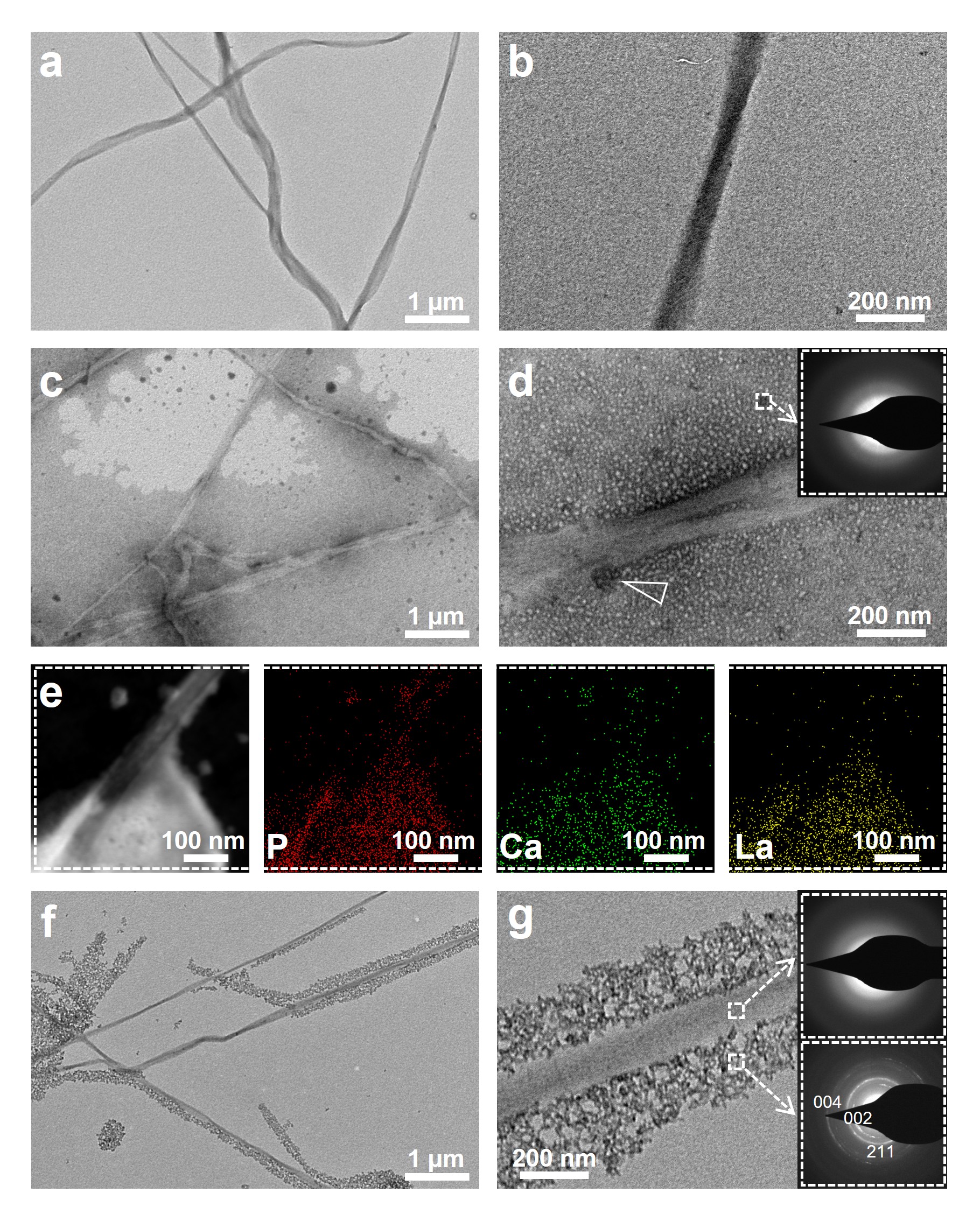


**Figure S7. TEM images with SAED patterns and elemental mapping of collagen fibrils after the treatment with Ca-La suspension followed by phosphate solution each for 1 h, and further incubation in artificial saliva for 4 d.** a,b) TEM images reveal that the collagen fibrils became darker after the treatment with Ca-La suspension for 1 h. c,d) TEM images with SAED pattern reveal that the electron density was significantly decreased within the collagen fibrils and numerous amorphous minerals (white arrowhead in “d”) were mainly intercepted outside the collagen fibrils after further treatment with phosphate solution for 1 h. e) The element mapping results of the collagen fibril in “c” show that these amorphous minerals outside of the collagen fibril were composed of phosphorus, calcium and lanthanum elements. f,g) TEM images reveal that the collagen fibrils failed to be mineralized and the minerals were almost generated outside the collagen fibrils after further incubation in artificial saliva for 4 d. SAED patterns (insert in “g”) indicate that no distinct crystal could be detected on the collagen fibril while the extrafibrillar minerals were La-HAp with distinct (002), (211) and (004) planes.


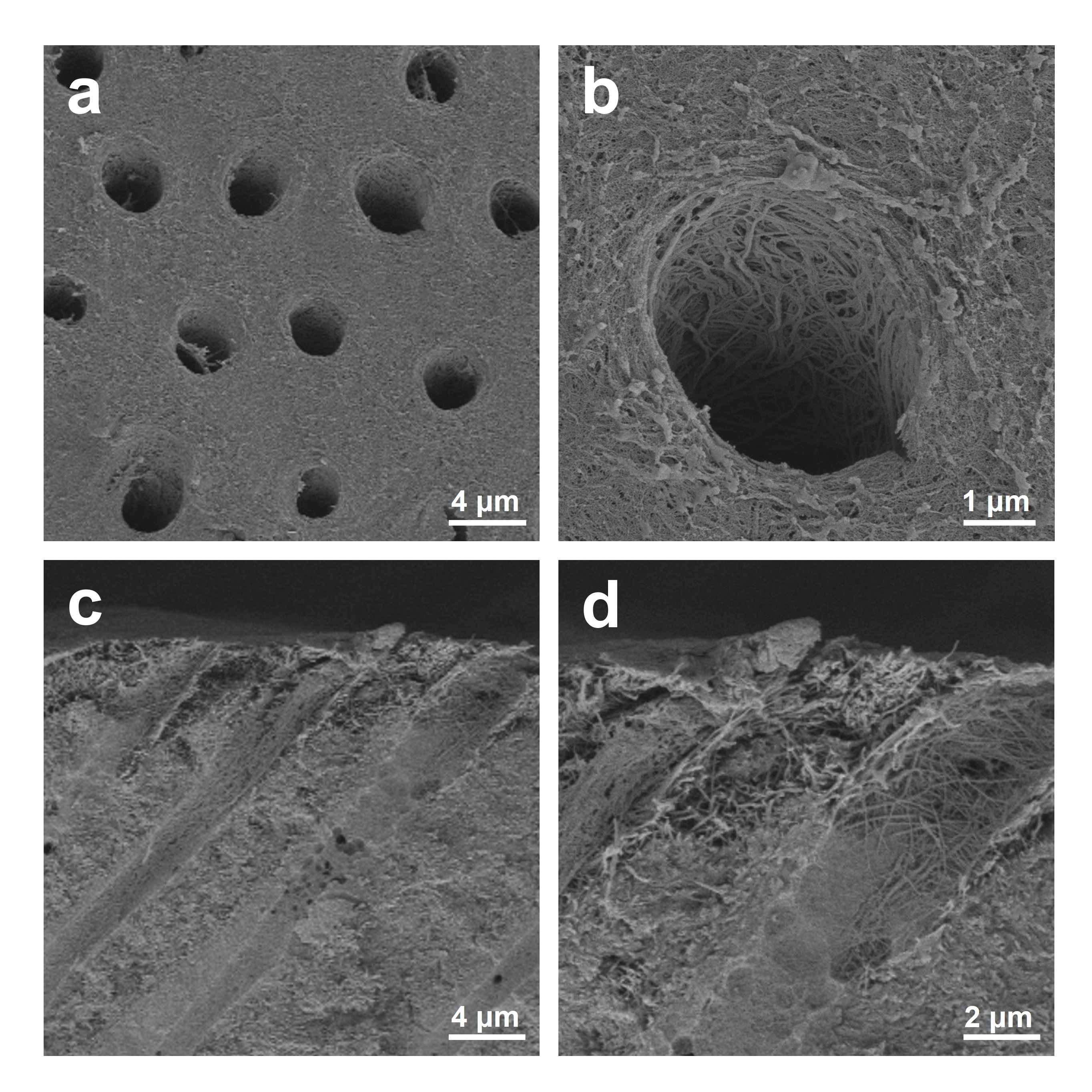


**Figure S8. The SEM images of DDM and exposure of DTs.** The etched dentin resulted in the exposure of DTs and a demineralized dentin layer of approximately 4-μm thickness.


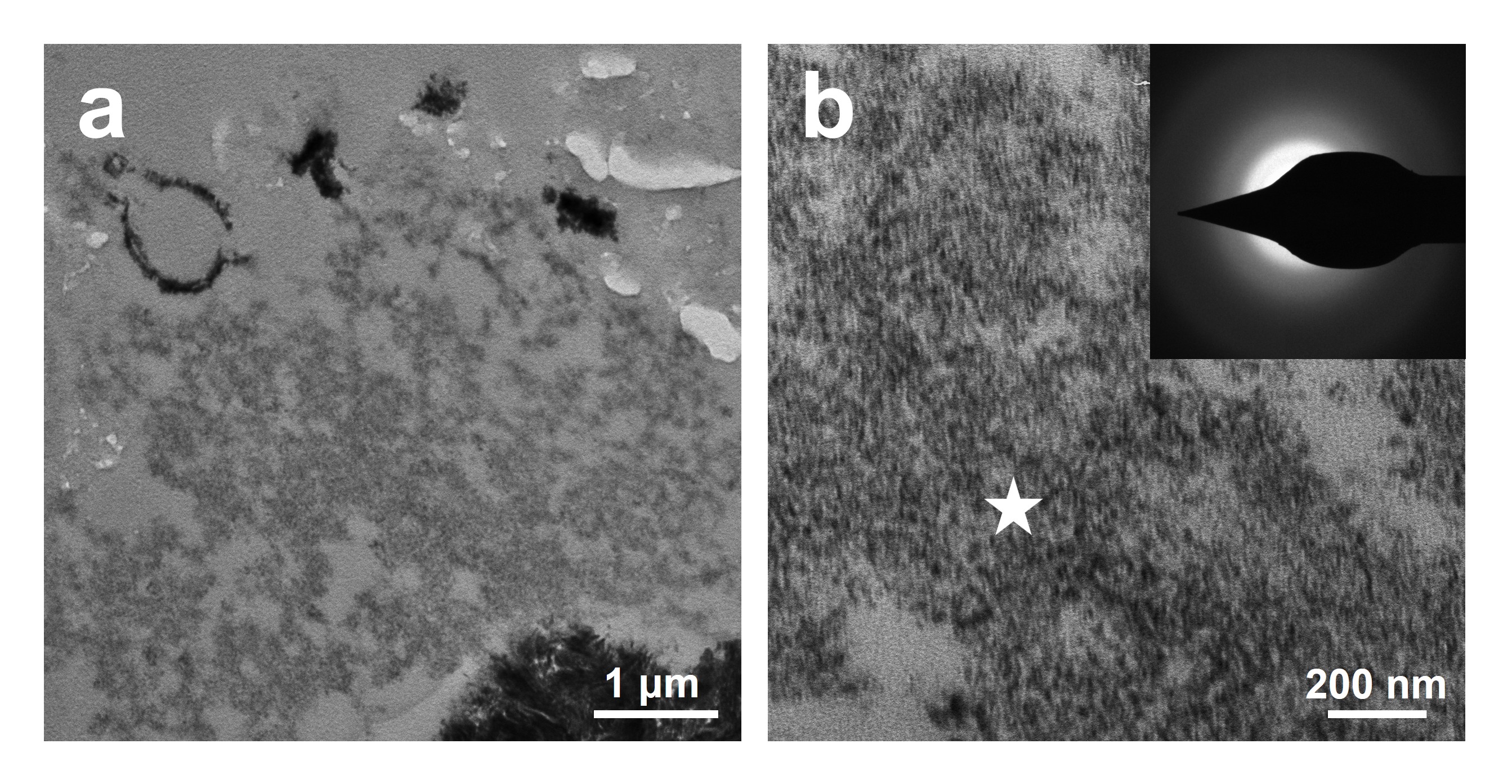


**Figure S9. TEM images with SAED pattern of the demineralized dentin after the treatment of PAsp-Ca-La suspension for 2 h and followed by phosphate solution for 2 h.** The formed minerals within the whole demineralized layer were still amorphous. The SAED pattern was obtained from the point marked with a white pentagram in “b”.


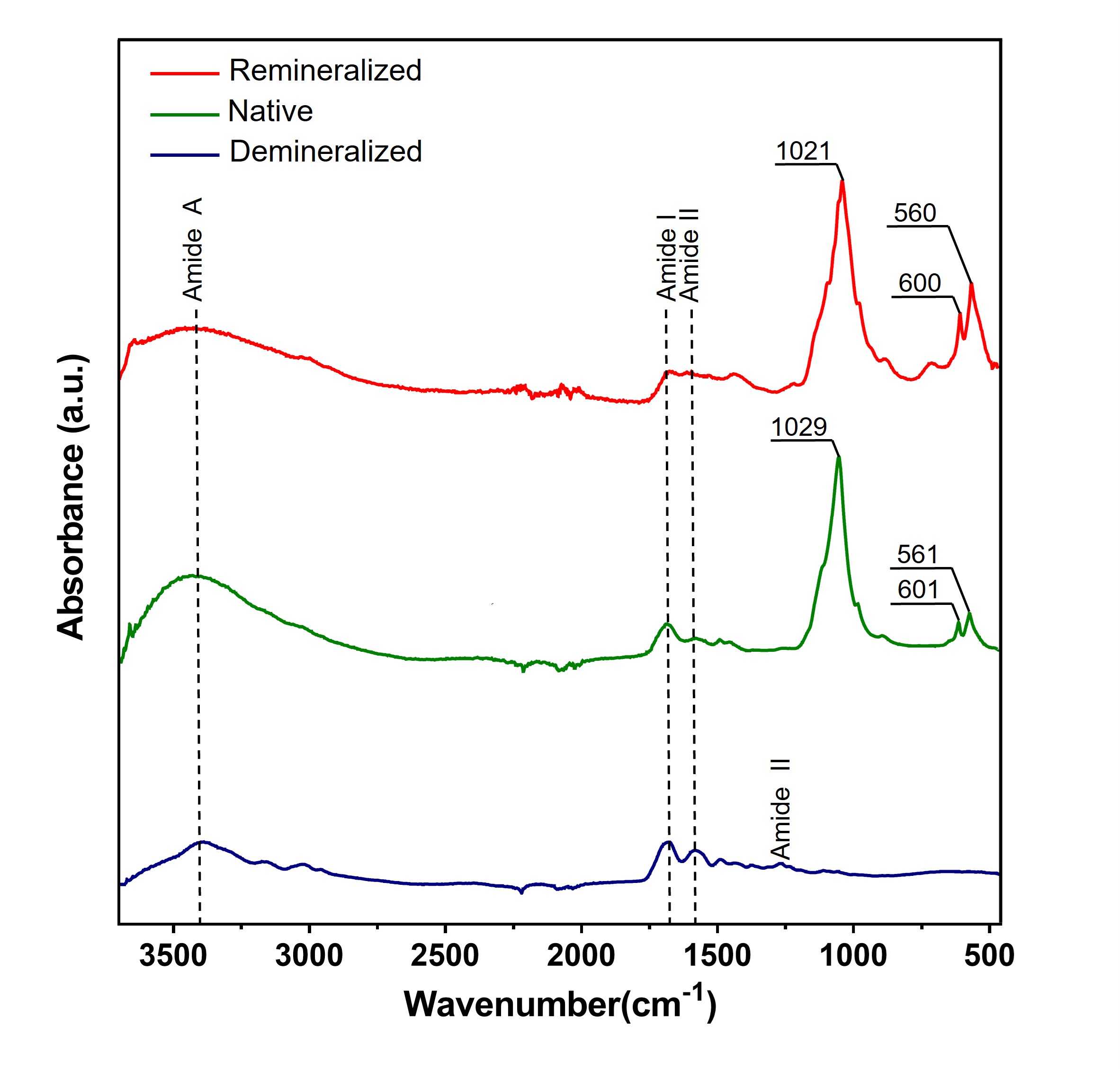


**Figure S10. ATR-FTIR spectra of the native, demineralized and remineralized dentin.** ATR-FTIR spectrum of the native dentin shows the characteristic absorbance bands corresponding to the amide bands of collagen fibrils and the phosphate groups of HAp. However, the characteristic absorption bands corresponding to the phosphate groups nearly vanished in the demineralized dentin. After sequential treatment with PAsp-Ca-La suspension and phosphate solution each for 1 h, and further incubation in artificial saliva for 4 d, the ATR-FTIR spectrum of the remineralized dentin exhibits characteristic absorption bands of phosphate groups. Specifically, the absorbance bands of phosphate groups in La-doped remineralized dentin shifts from the native dentin peaks of 1029, 601, and 561 cm^-1^ to 1021, 600, and 560 cm^-1^, respectively.

**
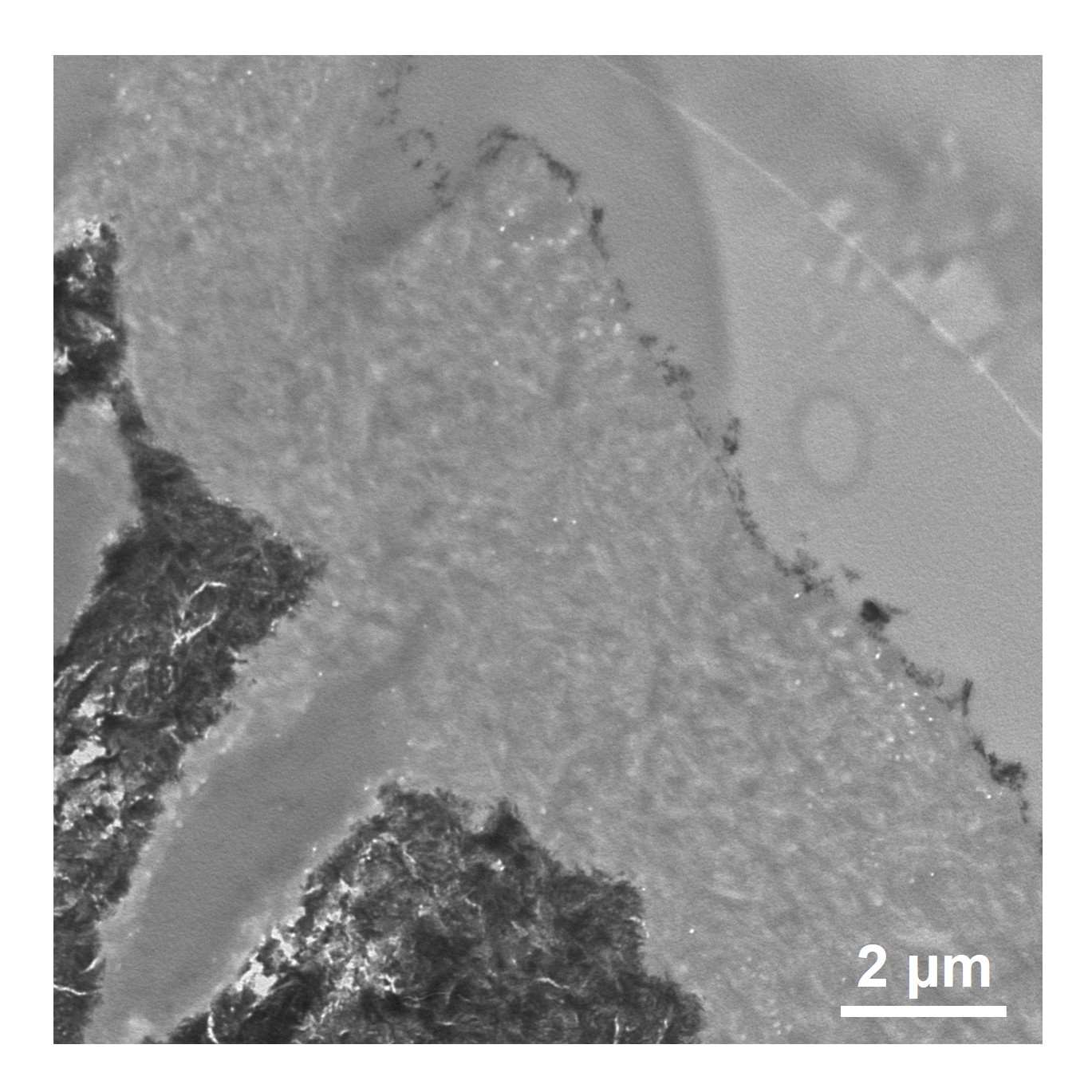
**

**Figure S11. TEM image of the demineralized dentin after incubation in artificial saliva for 7 d.** Although a few crystals were deposited on the dentin surface, no distinct mineral was observed in the DDM.


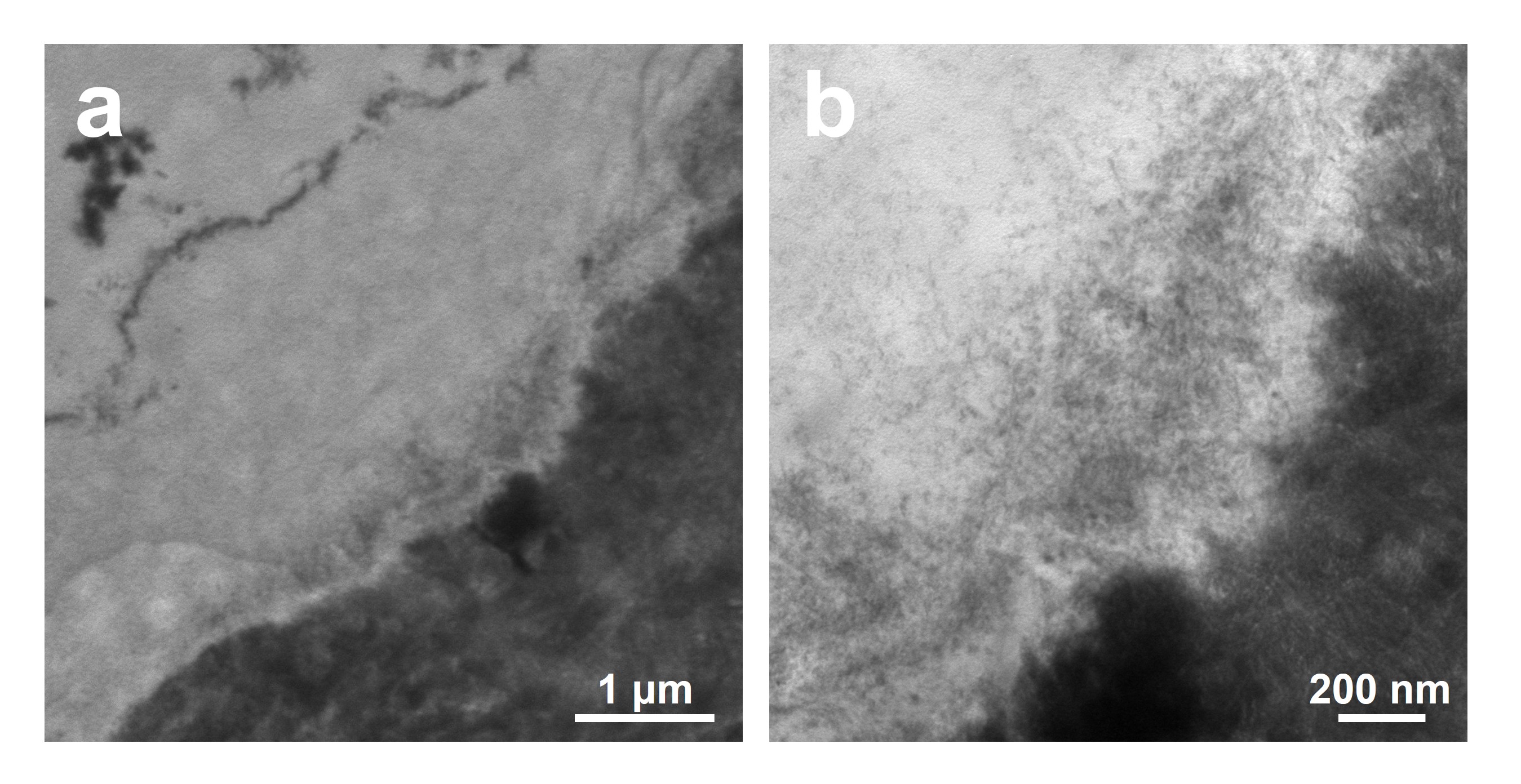


**Figure S12. TEM images of the demineralized dentin after the treatment of La-ACP solution for 7 d.** The minerals were formed on the dentin surface and in the bottommost DDM with an approximate thickness of 500 nm.


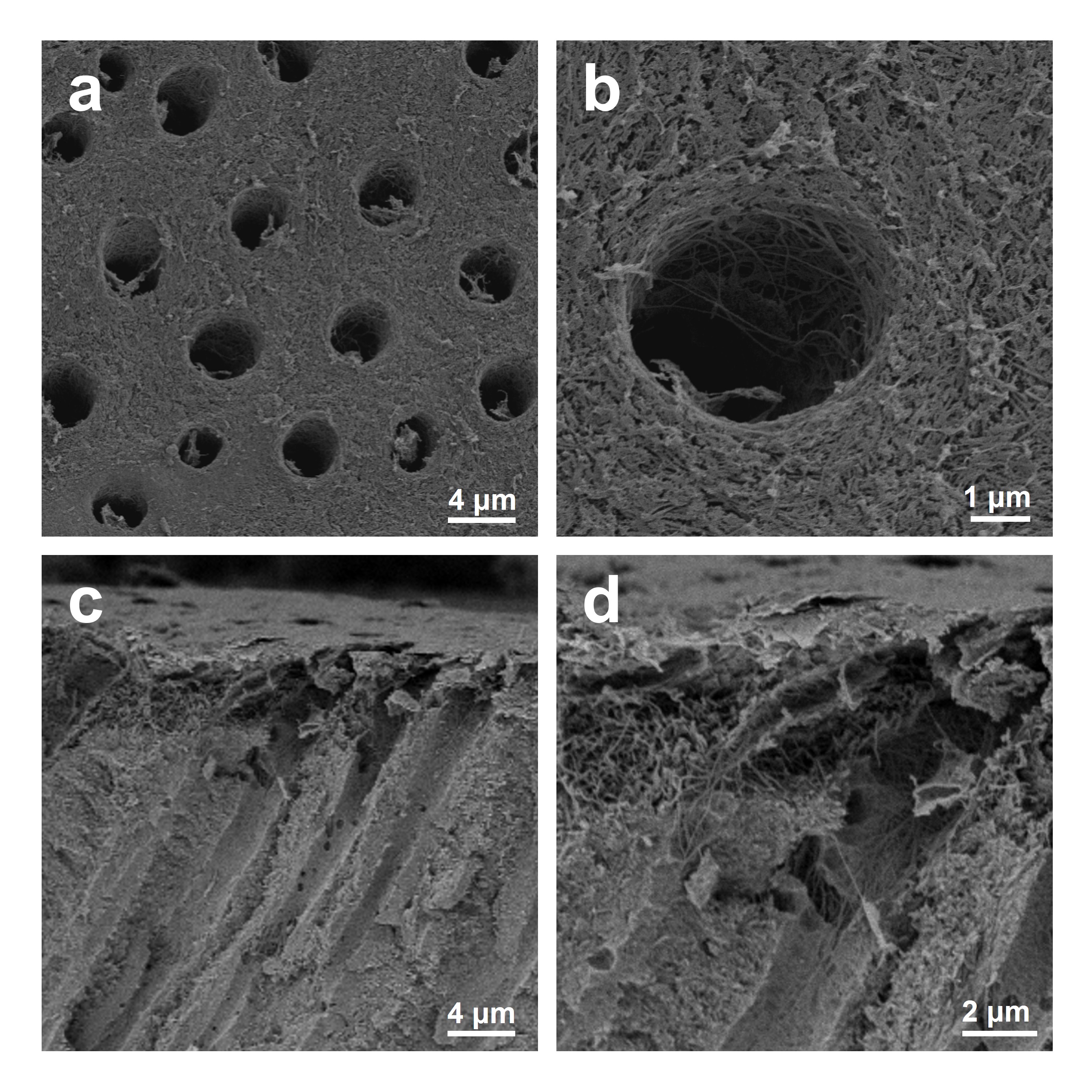


**Figure S13.** **SEM images of demineralized dentin that was only incubated in artificial saliva for 7 d revealed exposed DTs and DDM with** **naked collagen fibrils.**


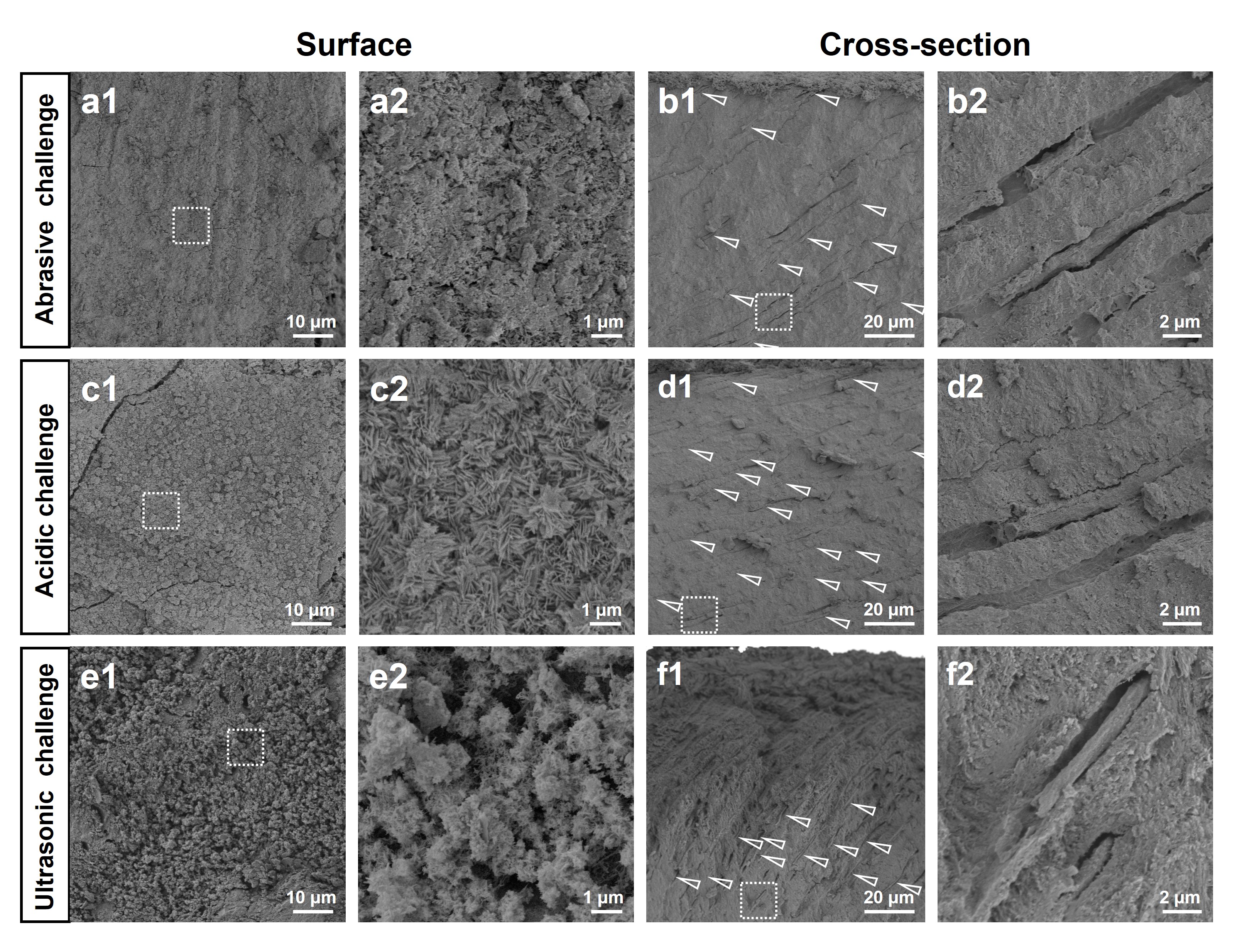


**Figure S14. The SEM images of the etched dentin disks after the treatment of the PCCP mineralization media following the abrasive (a,b), acidic (c,d), or ultrasonic (e,f) challenges.** “a2-f2” are the high magnification views of the white line box areas in “a1-f1”, respectively. White arrowheads in “b1”, “d1” and “f1” represent the occluding minerals within the DTs. a,b) After the abrasive challenge, tiny spaces among minerals could be detected on the dentin surface, and the DTs remained unexposed (a1,a2) and deeply occluded by dense crystals (b1,b2). c,d) After acidic challenge, a layer of crystals deposited on the dentin surface could be detected, and the DTs were not exposed (c1,c2), still occluded by dense minerals along the DTs (d1,d2). e,f) After ultrasonic challenge, the minerals were loosely distributed on the dentin surface without any exposure of DTs (e1,e2). The occluding minerals in the superficial area of the DTs were partly removed, but remained under the superficial dentin (f1,f2).


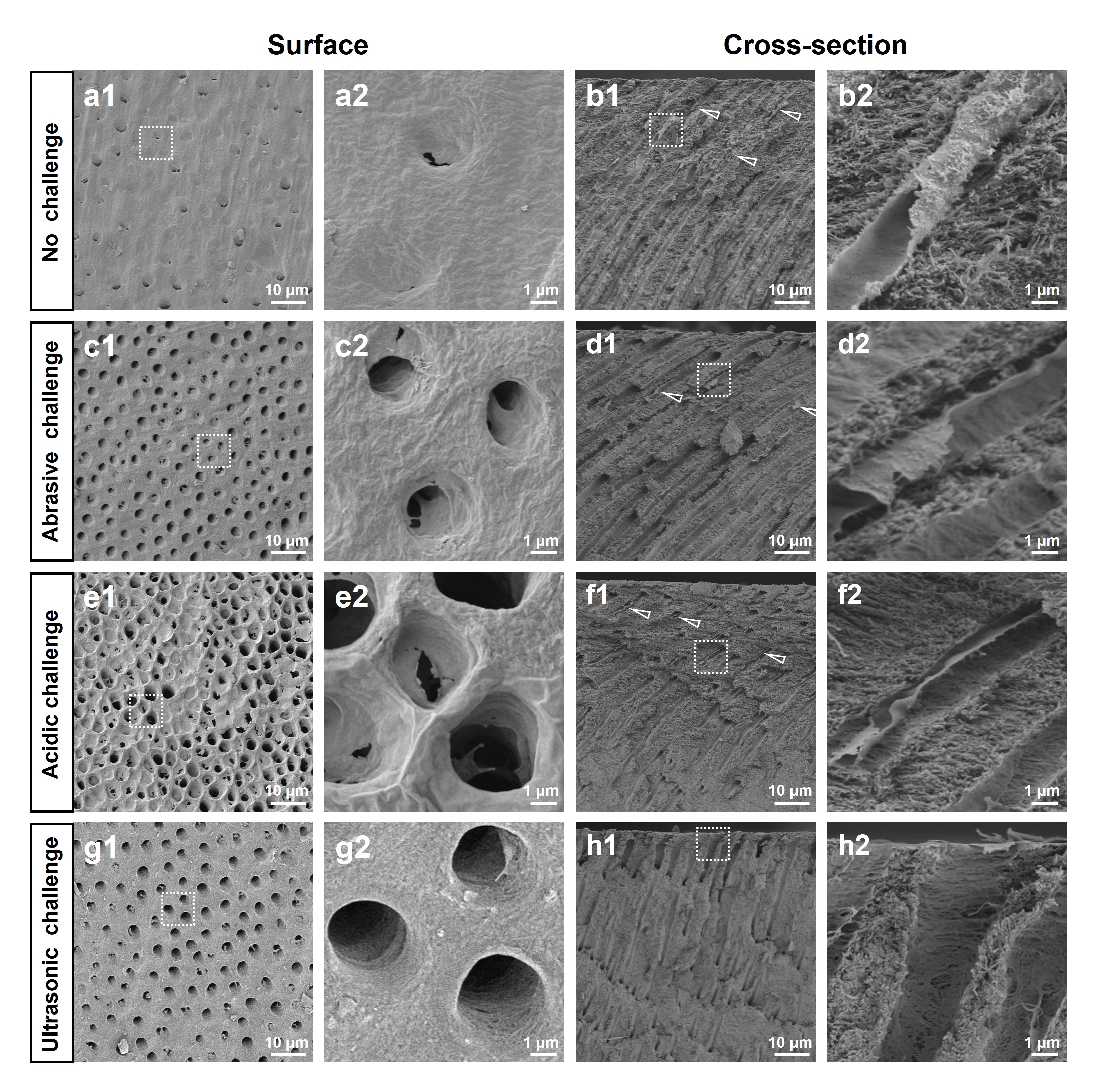


**Figure S15. The SEM images of the etched dentin disks after the treatment of Gluma desensitizer (a,b) following the abrasive (c,d), acidic (e,f), or ultrasonic (g,h) challenges.** “a2-h2” are the high magnification views of the white line box areas in “a1-h1”, respectively. White arrowheads in “b1”, “d1” and “f1” represent the occluding materials within DTs. a,b) After the treatment of Gluma desensitizer, some DT orifices were partly open (a1,a2), and some occluding materials with a hollow structure were closely adhered to the inner wall of the superficial DTs (b1,b2). c,d) After abrasive challenge, the majority of the DTs were exposed (c1,c2). The occluding materials were detected surrounding the superficial DTs (d1,d2). e,f) After acidic challenge, only few DT orifices were covered and, to some degree, deformed (e1,e2). The structure of occluding materials became looser within the DTs (f1,f2). g,h) After ultrasonic challenge, the DT orifices were almost completely patent (g1,g2), and the interior of the DTs was mostly empty (h1,h2).


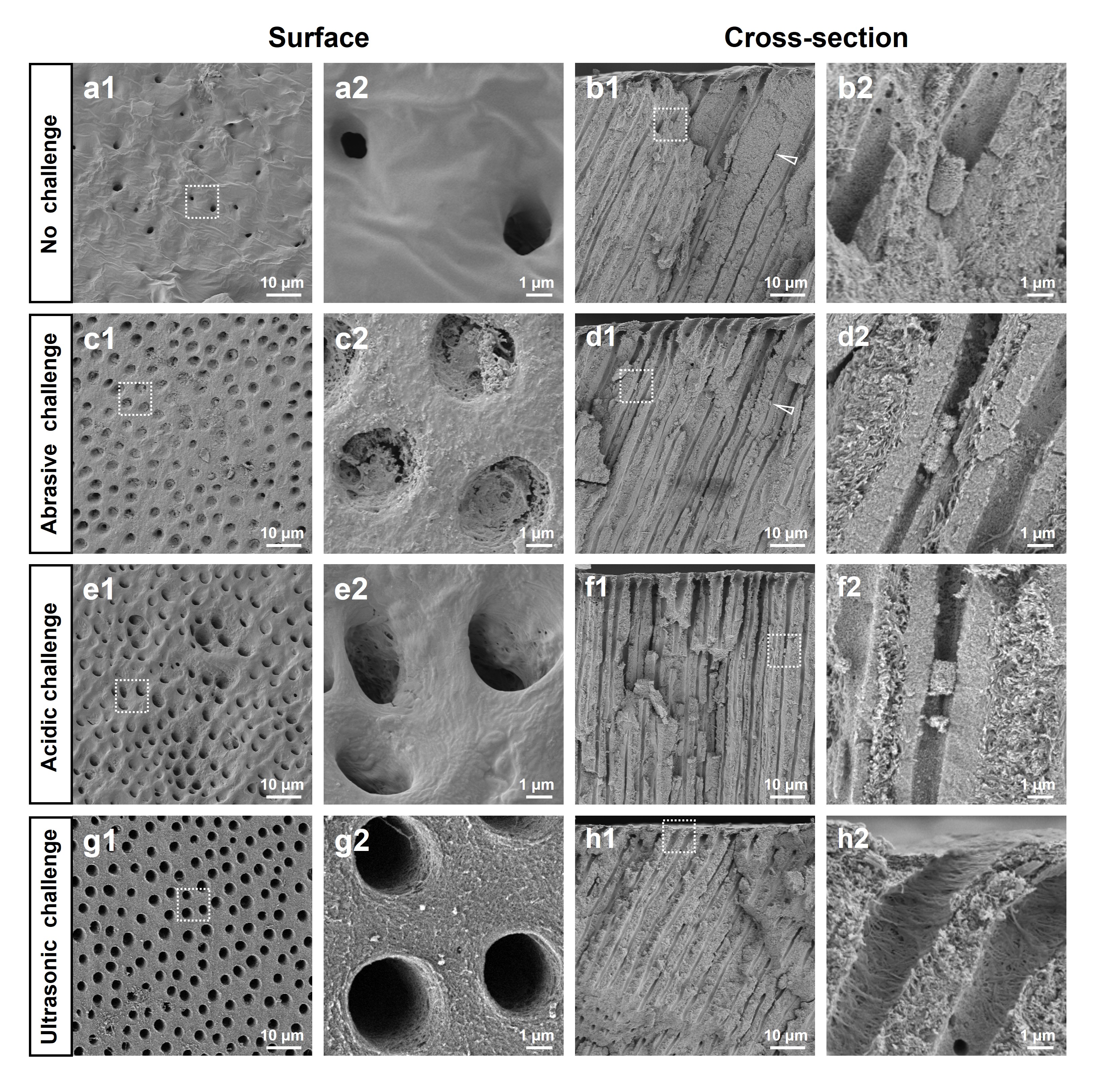


**Figure S16. The SEM images of the etched dentin disks after the treatment of Duraphat desensitizer (a,b) following the abrasive (c,d), acidic (e,f), or ultrasonic (g,h) challenges.** “a2-h2” are the high magnification views of the white line box areas in “a1-h1”, respectively. White arrowheads in “b1” and “d1” represent the occluding minerals within DTs. a,b) After the treatment of Duraphat desensitizer, most of the DT orifices were covered by a layer of varnish (a1,a2), and the minerals within the DTs were sparse and scattered (b1,b2). c,d) After abrasive challenge, the varnish on the dentin surface was mostly removed, with some DT orifices retaining mesh-like, loosely structured occluding materials inside (c1,c2). The columnar occluding minerals deposited within the DTs still could be detected (d1,d2). e,f) After acidic challenge, the orifices of the DTs were fully exposed (e1,e2). The sparse minerals deposited within the DTs still could be detected (f1,f2). g,h) After ultrasonic challenge, the DT orifices were completely patent (g1,g2) with hollow DTs (h1,h2).


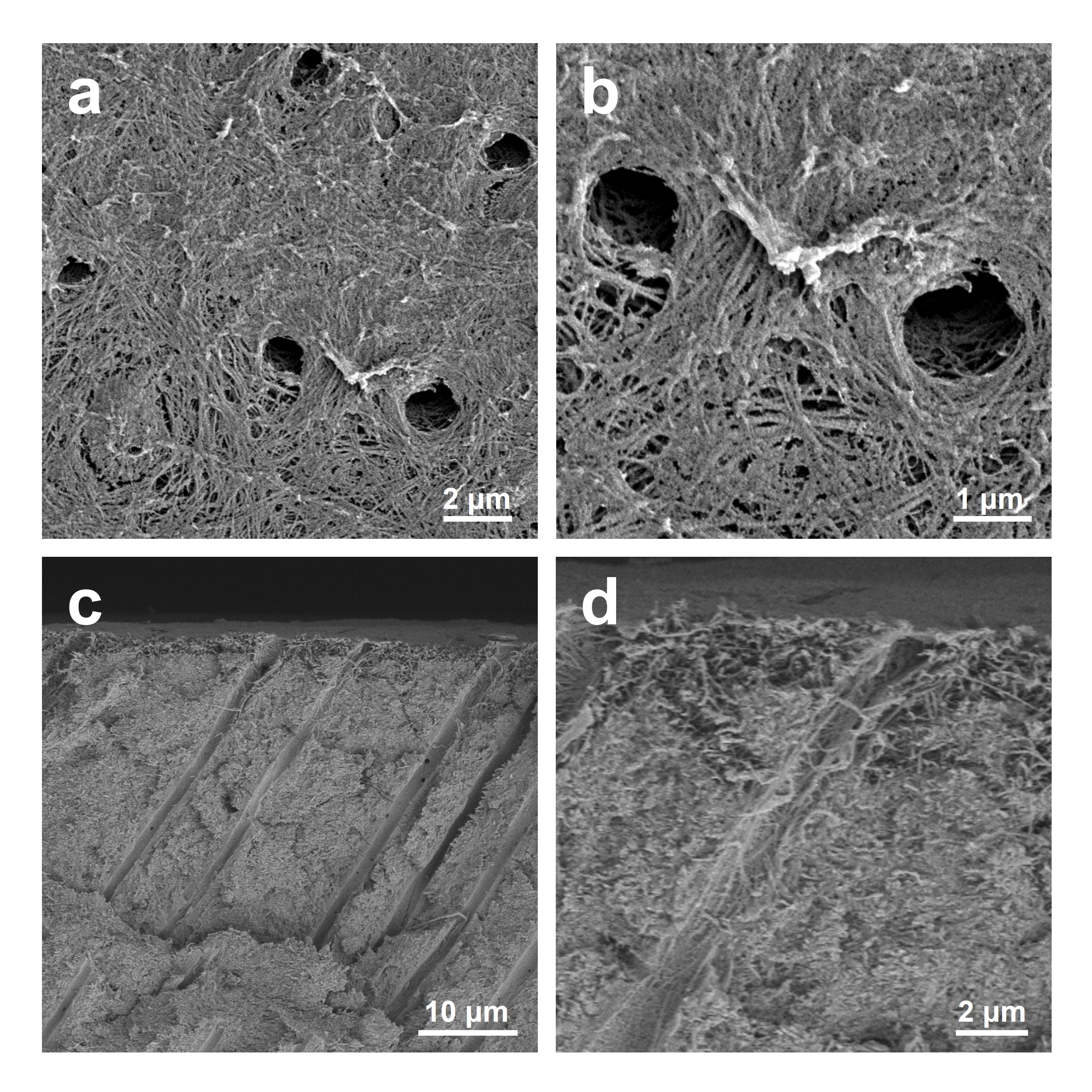


**Figure S17. SEM images of etched New Zealand rabbit incisor dentin after incubation in the oral cavity of rabbits for 7 d without any treatment.** SEM images show exposed of DTs in transverse section (a,b) and naked collagen fibrils in longitudinal section (c,d).


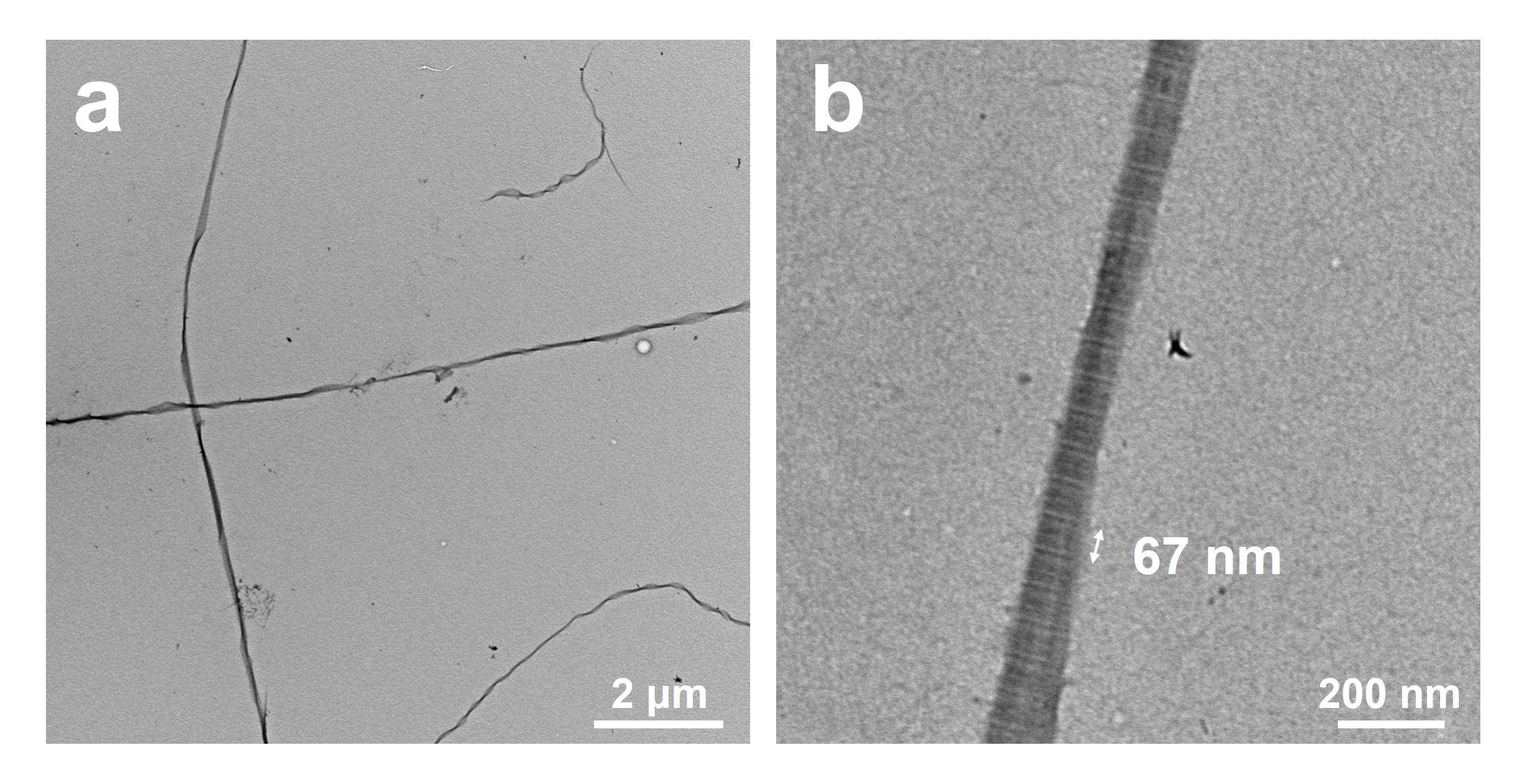


**Figure S18. TEM images of the reconstituted single-layer type I collagen fibrils stained with uranium acetate.** A typical period of 67 nm of type I collagen was observed in the high magnification (b).

**2. Materials and methods**

**2.1 Reconstitution of the single-layer type I collagen fibrils model on grids**

A 50 g mL^-1^ collagen solution was obtained by diluting 8.33 μL of rat tail type I collagen solution (3 mg mL^-1^) into 0.5 mL of buffer solution (50 mм glycine, 200 mм KCl, pH = 9.2) and then preserved at room temperature for 20 min. The grids loaded with 3 μL of collagen solution were incubated under the condition of saturated humidity at 37℃ for 12 h. After 1 h of incubation in a 0.05 wt% glutaraldehyde solution to facilitate cross-linking, the grids coated with collagen were rinsed with deionized water and ultimately retrieved.

**2.2 Preparation of the mineralized collagen gels**

300 µL of the commercial 3 mg mL^-1^ type I collagen was dropped onto a plastic film and assembled into fibrils after incubation in ammonia vapor for 4 h. Then, the collagen gels were incubated at 37℃ for 12 h and rinsed with deionized water until the pH became neutral. Subsequently, they were cross-linked with 0.05 wt% glutaraldehyde for 1 h and generally rinsed with deionized water. The collagen gels were sequentially soaked in the PAsp-Ca-La suspension and phosphate solution each for 1 h, with 5-min blot-drying after each soak. Subsequently, each specimen was immersed in 5 mL of artificial saliva (refreshed daily) for 4 d. Pure collagen gels served as controls. To compare with PCCP process without doping of La^3+^, the collagen gels were subjected to the treatment with PAsp-Ca suspension (5 g L^-1^-3.4 м) along with phosphate solution (2.04 м), following the same procedure. All the specimens were retrieved, washed with deionized water, and air-dried overnight in an electro-thermostatic blast oven at 37°C.

**3. Additional Tables**

**Table S1 Grading system for oral mucosa reactions**

| Reaction | Numerical grading |
| --- | --- |
| Erythema and formation | |
| No erythema | 0 |
| Very slight erythema (barely perceptible) | 1 |
| Well-defined erythema | 2 |
| Moderate erythema | 3 |
| Severe erythema (beet-redness) to eschar formation | 4 |
| Other adverse changes of the tissues should be recorded and reported. | |

**Table S2 Grading system for microscopic examination for oral mucosa tissue reaction**

| Reaction | Degree | Numerical grading |
| --- | --- | --- |
| Epithelium | Normal, intact  Cell degeneration or flatting  Metaplasia  Focal erosion  Generalized erosion | 0  1  2  3  4 |
| Leucocyte infiltration (per high power field) | Absent  Minimal (less than 25)  Mild (26 to 50)  Moderate (51 to 100)  Marker (greater than 100) | 0  1  2  3  4 |
| Vascular congestion | Absent  Minimal  Mild  Moderate  Marked with disruption of vessels | 0  1  2  3  4 |
| Oedema | Absent  Minimal  Mild  Moderate  Marked | 0  1  2  3  4 |
